# Completion and Compliance Rates for an Intensive mHealth Study Design to Promote Self-Awareness and Self-Care Among Care Partners of Individuals With Traumatic Brain Injury: Secondary Analysis of a Randomized Controlled Trial

**DOI:** 10.2196/73772

**Published:** 2025-08-21

**Authors:** Noelle E Carlozzi, Jonathan Troost, Wendy L Lombard, Jennifer A Miner, Christopher M Graves, Sung Won Choi, Zhenke Wu, Srijan Sen, Angelle M Sander

**Affiliations:** 1 Department of Physical Medicine and Rehabilitation, University of Michigan, 2800 Plymouth Road, Ann Arbor, MI, 48109, United States, 1 7347638917; 2 Department of Surgery, University of Michigan, Ann Arbor, MI, United States; 3 Survey Research Center, Institute for Social Research, University of Michigan, Ann Arbor, MI, United States; 4 Michigan Institute for Clinical and Health Research, University of Michigan, Ann Arbor, MI, United States; 5 Department of Pediatrics, University of Michigan, Ann Arbor, MI, United States; 6 Biostatistics, School of Public Health, University of Michigan, Ann Arbor, MI, United States; 7 Michigan Institute for Data and Artificial Intelligence in Society, University of Michigan, Ann Arbor, MI, United States; 8 Department of Psychiatry, University of Michigan, Ann Arbor, MI, United States; 9 H. Ben Taub Department of Physical Medicine and Rehabilitation, Baylor College of Medicine, Houston, TX, United States; 10 Brain Injury Research Center, TIRR Memorial Hermann, Houston, TX, United States

**Keywords:** caregivers, mobile apps, self-care, ecological momentary assessment, quality of life

## Abstract

**Background:**

Compliance rates for mobile health (mHealth) studies that involve intensive study designs are highly variable. Both person- and study-specific factors likely contribute to this variability. We were interested in understanding the impact that care partner characteristics and demographics have on study engagement, given that engagement is critical to the success of mHealth interventions.

**Objective:**

The primary objective of this report was to analyze the overall and component-specific completion and compliance rates for an intensive 6-month mHealth intervention (CareQOL app) designed to promote self-awareness and self-care among care partners of individuals with traumatic brain injury.

**Methods:**

This randomized controlled trial was designed to test the CareQOL app, an mHealth app designed to promote care partner self-awareness (through self-monitoring) and self-care (through personalized self-care push notifications). The study design consisted of a baseline assessment, a 6-month home-monitoring period that included 3 daily ecological momentary assessment (EMA) questions, monthly patient-reported outcome (PRO) surveys, continuous activity and sleep monitoring using a Fitbit, and 2 follow-up PRO surveys at 3 and 6 months posthome monitoring. Three participants withdrew prior to the initiation of the home-monitoring period, resulting in a final analytical sample size of 254. All participants had access to a self-monitoring dashboard (CareQOL app) that included graphical displays of the daily survey scores, as well as daily steps and sleep data from the Fitbit.

**Results:**

Overall compliance for the different aspects of the study was high. On average, the full-sample daily EMA PRO completion rate was 84% (SD 19%), Fitbit-based step count compliance was 90% (SD 21%), and Fitbit-based sleep duration compliance was 75% (SD 32%); there was no difference between the study arms for daily EMA PROs and Fitbit compliance rates. Completion rates for monthly and follow-up PRO surveys were even higher, with average end-of-month completion rates ranging from 97% to 100%, and follow-up completion rates of 95% for both time points. Again, these rates did not differ by study arm. The data were represented by 3 engagement groups: high-compliance—all data; high-compliance—PROs and steps only; and moderate PRO compliance—low Fitbit compliance. Group membership was predicted by both race (*P*<.001) and relationship to the care recipient (*P*=.001), but not by the other person-specific variables.

**Conclusions:**

The compliance rates for this intensive study design are consistent, but at the high end, with what has been reported previously in the literature for studies with shorter time durations. Except for race and relationship to the care recipient, person-specific factors did not appear to be significantly associated with the engagement group. As such, we anticipate that the high compliance rates observed in this study are likely due to several study-specific design elements that were used to encourage study engagement.

## Introduction

Survivors of traumatic brain injury (TBI) experience varying degrees of cognitive and motor function and require a range of acute, intermediate, and long-term supportive care based on the nature of their injury [1,2]. Much of this caring burden falls to informal (family) care partners, whose lives are upended by both dramatic changes in their loved one’s health and functioning and the sudden need to assume a medical caregiving role for which they may feel ill-equipped. The major lifestyle impacts and associated distress imposed by these changes increase these care partners’ risks for depression, anxiety, fatigue, social isolation, and sleep disturbance [3-7]. Not only do these problems negatively impact the care partners, but they also affect the health and well-being of the survivor of TBI who relies on them for care [3,5,8-11].

The time and energy demands of caring for someone with a TBI make it difficult for care partners to prioritize their own needs [12]. Thus, interventions targeting this group of care partners must be low burden, simple to use, and easy to incorporate into their day-to-day lives. Mobile health (mHealth) interventions are well-suited for this purpose due to widespread acceptance of mobile technologies and devices, as well as convenient access to mHealth-delivered content. In a recent systematic review of cognitive and behavioral digital health interventions among people with TBI and their care partners, mHealth interventions were found to be feasible, and all included studies demonstrated positive outcomes [13]. However, it is important to consider how differences in care partner characteristics and demographics may influence compliance rates for mHealth studies that involve ecological momentary assessments (EMAs), which have been shown to be highly variable.

In general, compliance for brief, intensive mHealth studies (ie, 2 wk or less) is moderately high, ranging from 57% to 87% [14-20]; these rates tend to progressively worsen as the study duration increases (ranging from 55% to 90% for studies lasting 2 to 4 weeks [21-30] and 77% for the single study we found that included a study duration of greater than 3 mo [31]). These high rates of variability are due to demographic factors [32], as well as person-specific [33] and protocol-specific factors [34]. For example, the literature has shown that racial and ethnic minorities tend to have lower compliance and no-show rates relative to their White or non-Hispanic counterparts [35-37]. Specifically, a recent review of barriers and facilitators to engaging minority populations in health care research identified mistrust (of health care professionals or systems, researchers, and research in general), cultural and language barriers, socioeconomic and logistical challenges, lack of research information and awareness, external influences (family and friends), and perceived bias from health care providers or researchers as the primary barriers to participation in research [38]. Furthermore, there are lower compliance rates among individuals with lower socioeconomic status, as well as individuals with less education [39,40]. Reasons for this include financial, time, and resource constraints associated with participation (eg, access to transportation, child and older people care, time off from work, or unanticipated costs), and disparities in health literacy [41-44]. Rates for age are mixed [45,46], but most studies show a positive association between age and compliance [47-54]. Furthermore, protocol-specific factors [34], which include survey length, study duration, and compensation amount, as well as compensation criteria, are highly variable across EMA studies and likely contribute to compliance rates (Table 1).

**Table 1.** Summary of compliance rates for published EMA^a^ studies.

Authors	Sample population	Sample size, n	Study length	EMA assessments, n	Average compliance rates for EMAs	Compensation
Jia et al [14]	Existing users of online food delivery	102 (53 in EMA group)	3 days	5 per day	72.5%	US $29 for completion of the entire study, US $9 if they completed 1/3 of the study, and US $19 for 2/3 of the study
Trang et al [15]	Men who have sex with men	46	7 days	6‐8 per day	61.8%	US $0.30 for each completed EMA
Solk et al [16]	People with breast cancer	75	10 days	4 per day	86%	US $100 for each of 3 check-ins, regardless of compliance
Kratz et al [17]	People with spinal cord injury and chronic pain	131	7 days	5 per day	81%	US $5 per day for days 1‐3, US $10 per day for days 4‐5, and US $20 per day for days 6‐7 of home monitoring
Smiley et al [18]	Gay and bisexual men	25	14 days	3 per day	57.3%	US $25 for <50% of EMAs completed; US $50 for ≥50% of EMAs completed
Ponnada et al [Bibr R19][19]	University students or staff	17	7 days	72 per day	87%	Not reported
Elbin et al [20]	Adolescents and young adults with concussion	116	7 days	4 per day	64%	Not reported
Tonkin et al [21]	Daily cigarette users	81	9 weeks	1 scheduled and 4 random per day	58% for random and 86% for scheduled	US $1 per assessment completed
Niznik et al [22]	Young adult cannabis and tobacco users	97	28 days	3 random per day	55%	US $20 per week for 4 weeks of IVR^b^ monitoring, an additional US $1 for each random assessment completed (maximum of US $3 per day), and a bonus of US $2 per week for completing assessments 6 of 7 days or US $5 per week for completing assessments for all 7 days
Juengst et al [23]	People with traumatic brain injury	20	8 weeks	2‐4 per day	73.4%	Not reported
Krohn et al [Bibr R24][24]	People with postpartum depression	26	6 weeks	2 per day	67%	Not reported
Slade et al [25]	College students	145	30 days	1 per day	78%	Not reported
Joo et al [26]	People with chronic pain with cannabis and opioid use	133	30 days	4 random prompts and 1 scheduled	89.7% for scheduled and 63.3% for randomly prompted	US $2 for completing at least one EMA each day, plus US $60 bonus if they achieved a 75% or greater EMA compliance rate over the study duration
Yang et al [27]	Men who have sex with men	16	4 weeks	3 random prompts and 1 morning prompt	74% of random prompts and 80.7% of morning prompts	US $50 every week for answering 80% of their alarms or US $25 every week for answering 60% of their alarms. They received no payments for answering less than 60% of their alarms or if their phone was uncharged
Beres et al [28]	People in Uganda	50	90 days	1 fixed and 1 random	66.5%	~US $30 for their time for responding to ≥50% of prompts
Sanjuan et al [29]	Pregnant women with prior trauma exposure	33	28 days	3 per day	74%	US $10‐40 for each weekly EMA download (dependent upon EMA completion rate), and US $25 extra for the final EMA download.
Laborde et al [30]	Older adults with knee osteoarthritis	27	~2 weeks	4 per day	83%	Not reported
Howard and Lamb [31]	Undergraduate alcohol drinkers	196	14 weeks	1.9 per day (13 per week)	76.5%	US $1 in Amazon cash for each survey; daily draws awarding US $25 in Amazon cash to one winner per day (Friday through Sunday; completion of at least one survey on the previous day to be eligible). After weeks 5 and 10, two unannounced US $5 bonuses to students who remained enrolled

a EMA: ecological momentary assessment.

b IVR: interactive voice response.

In our own work, we have demonstrated compliance rates at the high end of the above ranges. Specifically, in a 1-week study that included 3 daily EMA questions, we found an average response rate of 83% [55]. In another, longer study (3 mo duration), we found an average response rate of 90% for once-daily EMA questions, and 96% and 85% for daily step count data and sleep duration estimates, respectively (derived from continuous monitoring with a wearable device) [56]. We believe that these high compliance rates can be attributed, at least in part, to several study-specific design elements focused on fostering participant engagement (including survey brevity, monetary compensation, and app-specific customization features, as well as regular reminders following a 3-day lapse in responding [57].

Regardless of our previous findings in other care partner populations, care partners of individuals with TBI are a population that may potentially have difficulty engaging with EMA over longer time periods due to elevated rates of TBI in racial and ethnic minority groups, established racial and ethnic differences in TBI caregiving styles (Black care partners have been found to include more nonspouse relatives, spend considerably more time providing direct care, and care for significantly more disabled survivors than White care partners [58]), and the overall time commitment required of the caregiver role. We engaged a sample of care partners of individuals with TBI in an intensive study design that was of a longer duration (ie, a 12 mo study that included 6 mo of EMA PROs) [59]. In this study, participants completed a once-daily EMAs (3 items), wore a Fitbit, completed monthly surveys for 6 months, and completed follow-up surveys at 9 and 12 months. Previous examination of this dataset indicated high rates of compliance, with average completion rates of 84% (SD 19%) for once-daily EMA questions, and 90% (SD 21%) and 75% (SD 32%) for daily step count and sleep duration estimates, respectively (derived from continuous monitoring with a Fitbit) [59]. These study-specific race or ethnicity participation rates (20%) were comparable to or exceeded established US Census rates [60]. For the purposes of this analysis, we wished to explore these rates more closely and determine what, if any, additional demographic factors were related to compliance rates in our sample.

## Methods

### Participants

We enrolled care partners (ie, informal caregivers) of people living with TBI in this study between December 2020 and February 2023. Participants were recruited through two academic medical centers using clinical databases [61], site-specific registries, and community outreach. Recruitment often included contacting a person with a known TBI for their care partner referrals. Care partners needed to be at least 18 years of age, able to read and understand English, and caring for an adult at least 1 year postinjury who had sustained a medically documented complicated mild, moderate, or severe TBI. The injury must have occurred when the care recipient was aged 16 years or older. A care partner was defined as an individual who provided assistance to a person with a TBI (indicated by a response greater than 0 on the following eligibility rating question: On a scale of 0‐10, where 0 is “no assistance” and 10 is “assistance with all activities,” how much assistance does the person you care for require from you to complete activities of daily living due to problems resulting from his or her TBI?). Care partners were excluded if they did not have access to resources for participating in an mHealth intervention, including a personal mobile device capable of downloading the study apps for this study. Participants had to be willing to download the CareQOL and Fitbit apps to their device and be willing to complete all study assessments. In addition, we excluded professional, paid caregivers.

### Ethical Considerations

All study activities were conducted in accordance with institutional review board (IRB) approvals, and the study was registered with ClinicalTrials.gov (NCT04570930). The protocol, informed consent document, and all participant materials have received approval from IRBMED, which is the IRB of record for both data collection sites (IRBMED Multisite Application Approval HUM00181282; IRBMED University of Michigan Site Application Approval HUM00186921; IRBMED Baylor College of Medicine Site Application Approval SITE0000087; Baylor College of Medicine/Memorial Hermann IRB H-48478). All participants provided informed consent prior to the engagement in study activities. Participants were assigned a participant ID by the study team, which was used to avoid the inclusion of other identifying information. The electronic systems used to store the data collected in this study were secure systems with password protection and restricted access. Paper documents related to participation were stored in a locked cabinet or office. No names or other identifying information have been used in any report or publication of this study. This research was also covered by a Certificate of Confidentiality from the National Institutes of Health. Participants were compensated up to US $310 (US $20 each at baseline, 6 months, 3 months post, and 6 months post assessments; US $10 for each end of month survey for months 1‐5; and US $1 per day for each day that they had EMA or Fitbit data during the 6-month home monitoring period) and were allowed to keep the Fitbit. Compensation occurred monthly to encourage prompt responding. Participation was confidential, and study data were deidentified.

### Measures

A detailed description of the study protocol is provided elsewhere [62]. Briefly, participants completed a baseline assessment assessing demographic variables, proxy-reported measures of the care recipient’s functional and emotional status (Supervision Rating Scale [SRS] [63], Mayo-Portland Adaptability Inventory-Fourth Edition [64], and the Posttraumatic Stress Disorder Checklist for DSM-5 [65]), and 12 care partner self-reported health-related quality of life (HRQOL) PROs (Caregiver Strain [66,67], Caregiver-Specific Anxiety [67,68], Sleep-Related Impairment [69], Fatigue [69], Anxiety [69], Depression [69], Anger [69], Self-Efficacy-General [70], Positive Affect and Well-Being [69], Perceived Stress [70], Ability to Participate in Social Roles and Activities [69], and Global Health [71]). This was followed by a 6-month home monitoring period that included 3 care partner self-reported daily EMA PRO questions (single-item assessments of Caregiver Strain [66,67], Anxiety [69], and Depression [69]) and monthly self-reported (ie, PRO) surveys (again assessing the 12 HRQOL domains), as well as continuous monitoring of physical activity and sleep using a Fitbit. The 3- and 6-month follow-up HRQOL PROs were identical to the end-of-month PROs. In addition, a feasibility and acceptability survey was administered at the end of month 6 [56].

### Study Procedures

Participants were randomized to either a self-monitoring alone arm, which included completion of the daily EMA questions; baseline, monthly, and follow-up PRO surveys; and 6 months of continuous activity and sleep monitoring with a Fitbit, or to a self-monitoring plus self-care push notifications arm, which included self-monitoring plus self-care push notifications that involved a 50/50 chance each day of receiving a self-care prompt in addition to the other assessments. All participants had access to a self-monitoring dashboard (CareQOL app) that included graphical displays of the daily EMA scores, as well as daily step count and sleep duration data from the Fitbit. The proxy-reported measures were administered at baseline only through a REDCap (Research Electronic Data Capture; Vanderbilt University) survey, and self-reported PROs were administered through the CareQOL app at baseline, monthly, and both follow-ups. In addition, several electronic data capture and management platforms were used, including REDCap, CareQOL, Qualtrics, Fitbit, the University of Michigan Health Information Technology and Services server, and the Google Cloud.

### Statistical Analysis

First, we examined completion rates for daily EMA questions and monthly surveys, separately by study arm (calculated as the percentage of days with data over the number of days in the study for daily, monthly, and follow-up surveys), and then conducted a series of linear regression analyses to determine whether there were differences by study arm. Next, we examined the interrelationships among different types of completion (EMA questions, monthly survey responses) and compliance (Fitbit-based estimates of daily step count and sleep duration). We then used a backward selection process to determine which variables in the dataset related to compliance rates. For this analysis, we conducted a series of linear regression analyses to determine which variables (including demographic variables; baseline PROs, SRS, Mayo-Portland Adaptability Inventory-Fourth Edition, and Posttraumatic Stress Disorder Checklist for DSM-5; and feasibility and acceptability questions [assessed at 6 mo]) predicted completion or compliance rates.

Following this selection process, we used k-means clustering to identify latent (ie, “unobserved”) categorical subgroups of respondents based on their daily completion and compliance rates. The optimal number of clusters was determined based on assessment of model fit and parsimony (pseudo F statistic, approximate R-squared, cubic clustering criterion). Once the class number was determined, respondents were classified into latent classes based on maximum posterior probability. We also examined whether or not different descriptive variables (care partner age, care partner gender, care recipient age, care recipient gender, care partner race, care partner ethnicity, duration providing care, time spent caregiving, relationship to care recipient, work status, SRS score, PCL score, and functional ability of the care recipient) were able to predict the identified clusters.

## Results

Although 257 participants were initially randomized, 3 participants withdrew prior to the initiation of the study home monitoring period, resulting in a final sample size of 254 care partners of people with TBI. Of these 254 participants, 236 (92.9%) completed the 12-month study (n=118 for each of the individual study arms). Details describing the demographic data for the different study arms have been published previously (Carlozzi et al [59]). Table 2 provides an abbreviated summary of the demographic data for the full sample.

Overall compliance for the different aspects of the study was high (Tables3  4). Specifically, on average, the full-sample daily EMA completion rate was 84.5% (SD 19%), Fitbit-based step count compliance was 90.4% (SD 21%), and Fitbit-based sleep duration compliance was 74.9% (SD 32%); there was no difference between the study arms for daily completion and compliance rates (Table 3). Completion rates for monthly and follow-up surveys were even higher, with average end-of-month completion rates ranging from 97.2% to 100.0%, and follow-up completion rates of 95.1% for both 3-month and 6-month post time points; again, these rates did not differ by study arm (Table 4). Compliance rates were moderately correlated for the two Fitbit-based measures (*r*=0.65), and the magnitude of the correlations was less robust between Fitbit-based compliance data and the daily EMAs (*r*=0.38 between steps and EMAs and *r*=0.29 between sleep and EMAs).

A backward selection process indicated that: (1) several feasibility and acceptability questions related to ease, satisfaction, and burden of completing the EMAs predicted daily EMA completion rates; (2) race, relation to caregiver, and several of the feasibility and acceptability questions related to using the Fitbit predicted daily Fitbit-based step count compliance rates; and (3) race, relationship status, eligibility rating, and some of the feasibility and acceptability questions related to using the Fitbit predicted daily Fitbit-based sleep duration compliance rates (Multimedia Appendix 1). More specifically, positive perceptions about feasibility and acceptability were related to better completion and compliance rates for EMAs and monthly surveys, steps, and sleep; being Black was associated with lower Fitbit-based compliance rates for both steps and sleep; being a friend or other family member was associated with lower Fitbit-based compliance rates for daily steps; and being single and caring for someone with more functional deficits was associated with lower Fitbit-based compliance rates for sleep (Table 5).

**Figure 1.**
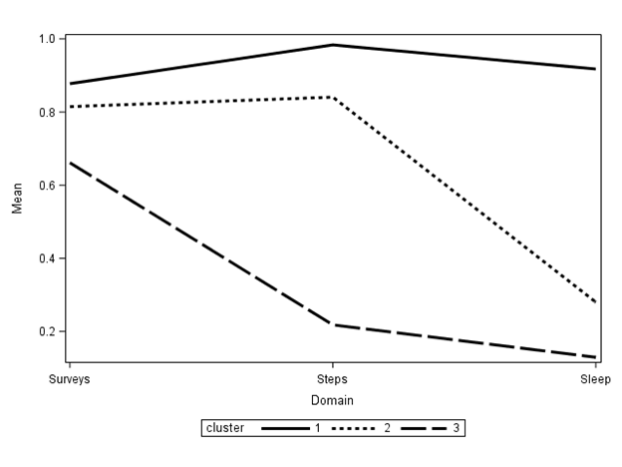
The solid line (cluster 1) represents a “high compliance group—all data” where compliance, across all data types (PROs, steps, and sleep) was high (72% of participants); the small dashed line (cluster 2) indicates a “high compliance group—PROs and steps only” where compliance was high for PROs and Fitbit-based steps, but not Fitbit-based sleep (22% of participants); and the large dashed line (cluster 3) indicates a “moderate PRO and low Fitbit compliance group” where monthly survey compliance was good, but not excellent, and Fitbit data were very low (7% of people).

Results from the cluster analysis did not indicate clear support for either a 2-, 3-, or 4-cluster model (Multimedia Appendix 2). The 2-cluster model indicated a “good” versus a “bad” compliance group, the 3-cluster model indicated: (1) a “high-compliance group—all data” cluster (cluster 1), where compliance across all data types (EMA and survey PROs, steps, and sleep) was high (n=182, 71.7% of participants); (2) a “high-compliance group—PROs and steps only” cluster (cluster 2), where compliance was high for EMA and survey PROs and Fitbit-based compliance rates for steps, but not for sleep (n=55, 21.7% of participants); and (3) a “moderate PRO compliance, low Fitbit compliance group” cluster (cluster 3), where monthly survey completion was good, but not excellent, and Fitbit compliance for both steps and sleep was low (n=17, 6.7% of people). The 4-cluster model further splits the “moderate PRO compliance, low Fitbit compliance group.” We elected to further explore the 3-cluster model (Figure 1) given the fact that this clustering was best aligned with the previous analyses (where we consider survey, sleep, and step compliance separately), as well as the overall number of people represented by each cluster (ie, we combined a data-driven approach with clinical interpretability for final cluster selection [72]).

Cluster membership was predicted by both race (*P*<.001), with a larger proportion of White caregivers relative to the other racial groups in cluster 1 (high compliance group—all data) relative to the other 2 clusters (high compliance group—PROs and steps only and moderate PRO and low Fitbit compliance group), and relationship to the care recipient (*P*=.001), with the moderate PRO and low Fitbit compliance group having a higher proportion of parents and a lower proportion of partners than the other two clusters, but not to other demographic factors or other characteristics (Table 6).

**Table 2.** Sample descriptive data (n=254).

	Value (n=254)
Age (years), mean (SD)	52.0 (14.7)
Age of person cared for (years), mean (SD)	43.0 (17.7)
Sex (female), n (%)	201 (79.1)
Sex of person cared for (female), n (%)	68 (26.8)
Race, n (%)	
American Indian or Alaska Native	3 (1.2)
Asian	10 (3.9)
Black or African American	36 (14.2)
More than 1 race	8 (3.1)
Native Hawaiian or Other Pacific Islander	1 (0.4)
White or Caucasian	195 (76.8)
Missing	1 (0.4)
Hispanic ethnicity, n (%)	41 (16.1)
Length of caregiving (years), mean (SD)	6.5 (5.7)
Relation to caregiver, n (%)	
Partner	104 (40.9)
Child	38 (15.0)
Parent	81 (31.9)
Sibling	20 (7.9)
Other family	3 (1.2)
Friend	3 (1.2)
Missing	5 (2.0)
Same household, n (%)	
Yes, all the time	182 (71.7)
Yes, but only a few days a week	13 (5.1)
No	59 (23.2)
Work status, n (%)	
Employed full time (at least 40 h/wk)	111 (43.7)
Employed part-time	29 (11.4)
Homemaker	17 (6.7)
Student	11 (4.3)
Retired	56 (22.0)
Retired early due to disability	2 (0.8)
Unemployed <1 year, and looking for work	4 (1.6)
Unemployed <1 year, not looking for work	1 (0.4)
Unemployed >1 year, looking for work	3 (1.2)
Unemployed >1 year, not looking for work	7 (2.8)
Unable to work or disabled	8 (3.1)
Other	5 (2.0)
How much assistance does the person you care for require from you to complete activities of daily living due to problems resulting from his or her TBI^a^? (0‐10), mean (SD)	6.3 (2.4)
Supervision Rating Scale	
Mean (SD)	4.0 (3.1)
Range	1‐12
Posttraumatic stress symptoms (PCL-5^b^)	
Mean (SD)	20.0 (15.6)
Range	0‐74
Time spent caregiving, n (%)	
1 to 2 hours per day or less	121 (47.6)
3 to 4 hours per day (ie, half of a working day)	54 (21.3)
5 to 8 hours per day (ie, full working day)	23 (9.1)
9 to 12 hours per day	13 (5.1)
>12 hours per day or round-the-clock care	43 (16.9)

a TBI: traumatic brain injury.

b PCL-5: Posttraumatic Stress Disorder Checklist for the *Diagnostic and Statistical Manual of Mental Disorders, Fifth Edition*.

**Table 3.** Daily compliance data for PROs^a^ and Fitbit data (n=254)^b^.

	Self-monitoring only		Self-monitoring plus self-care push notifications		Overall			
Assessment	Range	Mean (SD)	Range	Mean (SD)	Range	Mean (SD)	β (95% CI)	*P* value
Daily EMA^c^ questions	1‐100	86 (21)	4‐100	83 (18)	1‐100	84 (19)	−2.4 (−7.2 to 2.4)	.33
Daily steps	4‐100	90 (21)	2‐100	90 (21)	2‐100	90 (21)	0.00 (−5.3 to 5.3)	.99
Daily sleep	1‐100	74 (34)	1‐100	76 (31)	1‐100	75 (32)	2.4 (−5.7 to 10.4)	.56

a PRO: patient-reported outcome.

b Overall data are shown for comparative purposes only; it is reproduced from Carlozzi et al [59].

c EMA: ecological momentary assessment.

**Table 4.** Survey compliance data for baseline, end of month, and follow-up surveys (n=254).

	Self-monitoring only	Self-monitoring plus self-care push notifications	Overall	Chi-square (*df*)	*P* value
Baseline	100	100	100	—^a^	—
Month 1	100	100	100	—	—
Month 2	98	99	99	0.4 (1)	.56
Month 3	99	98	99	0.3 (1)	.57
Month 4	98	98	98	0.2 (1)	.66
Month 5	100	94	97	7.1 (1)	.008
Month 6	100	98	99	2.0 (1)	.16
3-month post	95	95	95	0.0 (1)	.98
6-month post	96	94	95	0.3 (1)	.57

a Not applicable.

**Table 5.** Variables with a statistically significant relationship with compliance rates.

	Daily EMAs^a^		Steps		Sleep	
	β (95% CI)	*P* value	β (95% CI)	*P* value	β (95% CI)	*P* value
Race				.045		<.001
Black	—^b^	—	−9.2 (−16.7 to −1.8)	.02	−31.2 (−42.2 to −20.0)	<.001
Other	—	—	−4.2 (−13.5 to 5.0)	.37	9.0 (−22.8 to 4.8)	.20
White	—	—	—	Reference	Reference	Reference
Relation to caregiver				.01	—	—
Child	—	—	−2.4 (−10.1 to 5.4)	.55	—	—
Friend	—	—	−30.6 (−54.7 to −6.6)	.01	—	—
Other family	—	—	−36.0 (−60.0 to −12.0)	.004	—	—
Parent	—	—	−2.5 (−8.6 to 3.5)	.41	—	—
Sibling	—	—	−4.1 (−14.1 to 5.9)	.42	—	—
Partner	—	—	Reference	Reference	Reference	Reference
Marital status (married vs single [reference])	—	—	—	—	18.1 (8.8 to 27.5)	<.001
Eligibility rating	—	—	—	—	−1.8 (−3.4 to −0.1)	.04
The Fitbit data were easy to sync with my phone (per 1 point on a 5-point Likert scale)	2.7 (0.1 to 4.6)	.005	6.2 (3.9 to 8.4)	<.001	7.1 (3.0 to 11.1)	<.001
I was confident using the Fitbit (per 1 point on a 5-point Likert scale)	2.7 (0.1 to 5.1)	.03	9.2 (6.4 to 12.1)	<.001	9.1 (3.9 to 14.2)	<.001

a EMA: ecological momentary assessment.

b Not applicable.

**Table 6.** Variables with statistically significant relationships with compliance rates.

	Cluster 1 (high compliance group—all data), mean (SD)	Cluster 2 (high compliance group—PROs^a^ and steps only), mean (SD)	Cluster 3 (moderate PRO and low Fitbit compliance group), mean (SD)	*P* value
Age (years; care partner)	49.9 (17.6)	52.8 (14.0)	49.9 (15.7)	.45
Age (years; care recipient)	36.9 (18.1)	44.1 (17.7)	41.0 (17.3)	.12
Sex				.50
Male (care partner)	2 (12)	41 (23)	10 (18)	
Female (care partner)	15 (88)	141 (77)	45 (82)	
Sex				.14
Male (care recipient)	9 (53)	137 (75)	40 (73)	
Female (care recipient)	8 (47)	45 (25)	15 (27)	
Race^b^				<.001
Black	4 (24)	12 (7)	20 (36)	
Other	2 (12)	14 (8)	6 (11)	
White	11 (65)	155 (86)	29 (53)	
Ethnicity				.21
Not Hispanic or Latino	15 (88)	148 (81)	50 (91)	
Hispanic or Latino	2 (12)	34 (19)	5 (9)	
Relation to caregiver^c^				.001
Child	3 (18)	26 (15)	9 (16)	
Friend	1 (6)	0 (0)	2 (4)	
Other family	2 (12)	1 (1)	0 (0)	
Parent	7 (41)	58 (33)	16 (29)	
Sibling	1 (6)	12 (7)	7 (13)	
Partner	3 (18)	80 (45)	21 (38)	
Living in the same household				.58
Yes, all of the time	12 (71)	134 (74)	36 (65)	
Yes, but only a few days a week	1 (6)	7 (4)	5 (9)	
No	4 (24)	41 (23)	14 (25)	
Length of time caregiving (years)	4.0 (3.4)	6.8 (5.9)	6.6 (5.35)	.09
Work status				.10
Employed full time (at least 40 h/wk)	5 (29)	80 (44)	26 (47)	
Employed part-time	2 (12)	22 (12)	5 (9)	
Homemaker	3 (18)	9 (5)	5 (9)	
Student	2 (12)	7 (4)	2 (4)	
Retired	3 (18)	45 (25)	8 (15)	
Retired early due to disability	0 (0)	1 (1)	1 (2)	
Unemployed less than 1 year, and looking for work	0 (0)	1 (1)	3 (5)	
Unemployed less than 1 year, not looking for work	0 (0)	1 (1)	0 (0)	
Unemployed for more than 1 year, looking for work	1 (6)	0 (0)	2 (4)	
Unemployed for more than 1 year, not looking for work	1 (6)	6 (3)	0 (0)	
Unable to work or disabled	0 (0)	7 (4)	1 (2)	
Other	0 (0)	3 (2)	2 (4)	
How much assistance does the person you care for require from you to complete activities of daily living due to problems resulting from his or her TBI^d^? (0‐10)	6.8 (2.51)	6.1 (2.44)	6.7 (2.22)	.13
Supervision Rating Scale	5.2 (3.9)	4.0 (3.1)	3.7 (2.9)	.47
Posttraumatic stress symptoms (PCL-5^e^)	20.9 (16.6)	18.9 (14.6)	23.3 (17.9)	.23
Time spent caregiving				.21
1 to 2 hours per day or less	4 (24)	93 (51)	24 (44)	
3 to 4 hours per day (ie, half of a working day)	3 (18)	38 (21)	13 (24)	
5 to 8 hours per day (ie, full working day)	3 (18)	14 (8)	6 (11)	
9 to 12 hours per day	1 (6)	11 (6)	1 (2)	
>12 hours per day or round-the-clock care	6 (35)	26 (14)	11 (20)	
Functional status of the person with TBI (MPAI-4^f^)	47.9 (20.31)	46.7 (17.49)	46.5 (20.60)	.78

a PRO: patient-reported outcome.

b One participant in cluster 2 was missing race.

c Five participants in cluster 2 were missing relationship to caregiver.

d TBI: traumatic brain injury.

e PCL-5: Posttraumatic Stress Disorder Checklist for DSM-5.

f MPAI-4: Mayo-Portland Adaptability Inventory-Fourth Edition.

## Discussion

### Principal Findings

Overall, there were high rates of compliance for a 6-month intensive home monitoring protocol that involved daily EMA ratings of HRQOL and continuous monitoring of physical activity and sleep by a Fitbit. Average compliance rates were highest (90%) for activity (step) monitoring (ie, wearing the Fitbit during the day), followed by EMA completion (84%), and lowest (75%) for sleep monitoring (ie, wearing the Fitbit overnight). In addition, the completion rates for the monthly HRQOL surveys were also high (ranging from 92% to 98%). Compliance and completion rates did not differ by study arm. There was only a moderate relationship between wearing the Fitbit during the day versus at night, and there was a less robust (ie, small) relationship between EMA completion rates and Fitbit compliance rates. These rates are consistent, at the high end, with what has been reported previously in the literature for studies with shorter time durations [14-31,33]. The data on compliance rates for longer study durations in the literature, such as we report from this study, is sparse, but would be expected to be lower than what we found here, given typical patterns of decline in compliance rates with longer study duration.

We were also interested in better understanding the participant-specific variables (eg, personality, comfort with technology, and wearable devices [33]) that might have impacted compliance rates. Not surprisingly, we found that positive perceptions about feasibility and acceptability were related to better compliance rates for EMA questions, surveys, steps, and sleep. This finding is consistent with the primary findings of this study, which showed that participants who were more positive about the study itself (regardless of study arm) were more likely to show HRQOL improvements [59], as well as those reported in the general literature that showed compliance rates were higher among participants who found the research study to be more favorable [73,74]. We also found that demographic variables impacted compliance. We found that being Black was associated with lower compliance rates for Fitbit data (sleep and steps), but not for EMA data. Historically, Black participants are underrepresented in research [75,76], and consistent with our findings, they have higher rates of missing data than their White counterparts [77-83]. Furthermore, being a friend or other family member of the person with TBI (vs a spouse or adult child of the person with TBI) was associated with lower compliance rates for Fitbit daytime wear data (steps). To our knowledge, while some meta-analytic work has examined caregiver populations (eg, caregivers of people living with dementia [84]), this work has not examined caregiver type in consideration of the differential factors that might influence missing data rates. Given this, we postulate that nontraditional caregivers (ie, friends and other family members) may feel less obligated to provide care than those in more traditional caregiver groups (ie, spousal and adult child caregivers). Future work to better understand these relationships, how they influence care, and how this may be related to study compliance rates is needed. We also found that being single or caring for someone with more functional deficits was associated with lower Fitbit nighttime wear data (sleep). While we are unaware of work that looks explicitly at these factors and nighttime compliance rates with wearables, we hypothesize that these types of caregivers may already be experiencing fragmented or disturbed sleep and therefore are more likely to find nighttime Fitbit wear uncomfortable and prohibitive [85].

While there were a handful of person-specific factors that contributed to compliance, our overall high rates of compliance for the different study elements emphasize the need for a focus on study-specific design elements that can help to mitigate these person-specific factors. For example, in this study, we postulate that the brevity of overall assessments, customization of administration windows for the EMA questions, and regular reminders following a 3-day lapse in responding may also have contributed to the higher response rates. Study staff completed regular checks for completion of the EMA questions and the presence of daily step and sleep data from the Fitbit and monthly surveys. In instances where participants were missing greater than 3 days’ worth of EMA or Fitbit data, we contacted those participants directly. In addition, automatic reminders for completion of the EMA questions and surveys were sent via the CareQOL app, and study staff contacted participants at least once per month during the home monitoring period to foster engagement. Additionally, we provided monetary compensation for the different elements of the study, including separate payments of US $20 for completing the baseline and 3-month and 6-month postmonitoring period follow-up surveys; US $10 compensation for completion of the monthly surveys during the home monitoring period (with the exception of the final monthly survey, for which participants were paid US $20); and US $1 per day for daily completion of the EMA questions or any daily data from wearing the Fitbit (either day or night). Participants could also keep the study-provided Fitbit after they completed the study. Participants were also able to customize what time they received their three EMA questions each day and were offered different wristband options for the Fitbit to maximize their comfort and style preferences.

These proposed factors align with literature that identifies a number of protocol-specific factors (eg, total number of questions, study duration, compensation rates, and criteria for compensation [34]) that appear to be characteristic of different study designs (Table 1).

Furthermore, when trying to maximize engagement for underrepresented groups, adopting a personalized approach to recruitment (eg, build rapport and use culturally sensitive communication styles), providing culturally appropriate financial incentives (eg, reimburse for travel costs), reducing language barriers (eg, representative research teams and culturally or linguistically appropriate study materials and communication strategies), engaging community champions in the recruitment process, addressing accessibility and logistical barriers to participation (eg, flexibility with location, timing, childcare, and transportation), participating in the community, and building cultural competence of the study team are strategies that have been shown to foster research participation and compliance among these groups [38,86].

Finally, we explored whether there were meaningful subgroups for different patterns of compliance rates. To this end, we found a “high compliance group—all data” that was compliant with all of the different study elements; a “high compliance group—PROs and steps only” where compliance was high for EMA and survey PROs and Fitbit-based compliance rates for steps, but not for sleep; and a “moderate PRO compliance, low Fitbit compliance group” where monthly survey completion was good, but not excellent, and Fitbit wear-time data were low. These subgroups were predicted by race and relationship to the care recipient, but not to other demographic, clinical, or behavioral characteristics, once again supporting the premise that study-specific enhancements to foster engagement would be beneficial for these groups.

### Limitations

While these results support the feasibility of care partner participation in studies that use intensive study designs (including EMAs over a 6-mo period, wearing of a wrist-worn device that provides continuous monitoring, and completion of end-of-month surveys), it is also important to acknowledge several study limitations. For example, while we have postulated about the reasons the observed completion rates were so high in this study, we did not systematically assess the impact that any of these factors had; future work is needed to explore the impact of factors, such as compensation and survey length, on participant completion and compliance rates. In addition, many participants in this sample did not endorse poor HRQOL at baseline, nor high levels of supervision required, nor high levels of assistance with activities required. This could mean that this sample is not experiencing high levels of strain as we had anticipated, and therefore, they may have been higher functioning than the general care partner population, such that they had less room for improvement, or they had more capacity or time to complete the intensive study activities. In addition, there was no clear front-runner for the clustering analyses. As such, we elected to explore the model that we felt best represented the data, but we acknowledge that this selection might represent an overfitting of the data.

### Conclusions

Overall, the results from this study indicated that although person-specific factors influence completion and compliance rates, it is still reasonable to expect high rates of compliance with an associated thoughtful study design that uses flexibility, tailoring, and financial incentives. Teasing apart the differential impact of the different study design elements on study participation will be a focus of future work. Furthermore, consistent with other literature, we found that disadvantaged groups (such as racial or ethnic minorities, single, or nontraditional caregivers) were more likely to have higher rates of missing data than their majority counterparts, further exemplifying the need for more focused work on understanding the reason for these lower rates and using methods to improve compliance among these groups.

## Supplementary material

10.2196/73772 Multimedia Appendix 1 Linear regression analyses (each cell indicates the *P* value for a separate model; italics indicate *P*<.05)

10.2196/73772 Multimedia Appendix 2 Assessment of model fit and parsimony.

10.2196/73772 Checklist 1 CONSORT-EHEALTH (Consolidated Standards of Reporting Trials of Electronic and Mobile Health Applications and Online Telehealth) checklist.

## Abbreviations

EMA: ecological momentary assessment
HRQOL: health-related quality of life
IRB: institutional review board
mHealth: mobile health
PRO: patient-reported outcome
REDCap: Research Electronic Data Capture
SRS: Supervision Rating Scale
TBI: traumatic brain injury
