# Supplementary material for: Completion and Compliance Rates for an Intensive mHealth Study Design to Promote Self-Awareness and Self-Care Among Care Partners of Individuals With Traumatic Brain Injury: Secondary Analysis of a Randomized Controlled Trial
Source: JMIR Mhealth Uhealth. 2025 Aug 21;13:e73772. doi: 10.2196/73772 (PMC12370270; doi:10.2196/73772)
Supplement: Multimedia Appendix 1 [file mhealth-v13-e73772-s001.docx]

|  | Beta [95% CI] | *p* | |
| --- | --- | --- | --- |
| EMAs | | |  |
| Age (care partner) | 0.00 [-0.00, 0.00] | 0.16 | |
| Age of person cared for | 0.00 [-0.00, 0.00] | 0.64 | |
| Sex (care partner) | 0.04 [-0.01, 0.08] | 0.10 | |
| Sex of person cared for | 0.02 [-0.02, 0.06] | 0.34 | |
| Race (care partner) |  | 0.49 | |
| Black vs. White | -0.03 [-0.09, 0.02] | 0.23 | |
| Other vs. White | -0.01 [-0.07, 0.06] | 0.86 | |
| Ethnicity (care partner) | 0.04 [-0.01, 0.09] | 0.10 | |
| Length of caregiving | 0.00 [-0.00, 0.01] | 0.29 | |
| Relation to care recipient |  | 0.047 | |
| Parent vs. Other | 0.02 [-0.03, 0.07] | 0.35 | |
| Partner vs. Other | 0.06 [0.01, 0.10] | 0.02 | |
| Same household as care recipient (Yes vs. No) | -0.00 [-0.05, 0.04] | 0.88 | |
| Relationships status (care partner) (Married vs. Not Married) | 0.04 [-0.00, 0.09] | 0.06 | |
| Work status (care partner) (Full-time vs. Other Categories) | -0.00 [-0.04, 0.03] | 0.84 | |
| Eligibility rating (care partner) | -0.00 [-0.01, 0.01] | 0.94 | |
| Supervision rating scale | -0.00 [-0.01, 0.00] | 0.11 | |
| PCL5 | -0.00 [-0.00, 0.00] | 0.98 | |
| Time spent caregiving (≥ 4 hours vs. < 4 hours) | -0.03 [-0.07, 0.01] | 0.11 | |
| MPAI4 | -0.00 [-0.00, 0.00] | 0.70 | |
| The instructions for the Fitbit^®^ set-up were easy to understand | 0.01 [-0.01, 0.03] | 0.38 | |
| The Fitbit^®^ was easy to set up | 0.01 [-0.01, 0.03] | 0.48 | |
| The Fitbit^®^ was easy to use | 0.02 [-0.01, 0.04] | 0.14 | |
| The Fitbit^®^ was comfortable to wear | 0.01 [-0.01, 0.03] | 0.194 | |
| The Fitbit^®^ data was easy to sync with my phone | 0.03 [0.01, 0.05] | 0.005 | |
| I was confident using the Fitbit^®^ | 0.03 [0.00, 0.05] | 0.03 | |
| The CareQOL app was easy to set up | 0.01 [-0.02, 0.04] | 0.41 | |
| The CareQOL app was easy to use | 0.02 [-0.01, 0.05] | 0.27 | |
| I was confident using the CareQOL app | 0.02 [-0.01, 0.04] | 0.27 | |
| I am confident that I was using the CareQOL app correctly | 0.02 [-0.01, 0.05] | 0.11 | |
| The daily questions on the app were easy to understand | 0.01 [-0.03, 0.04] | 0.61 | |
| The daily questions on the app were easy to answer | 0.02 [-0.01, 0.05] | 0.14 | |
| Answering the daily questions fit easily into my routine | 0.07 [0.04, 0.09] | <.0001 | |
| The monthly surveys on the app were easy to understand | 0.04 [0.01, 0.07] | 0.018 | |
| The monthly surveys on the app were easy to answer | 0.03 [0.00, 0.06] | 0.036 | |
| Answering the monthly surveys fit easily into my routine | 0.04 [0.01, 0.06] | 0.004 | |
| The intervention prompts had a positive impact on my health | 0.02 [-0.00, 0.05] | 0.10 | |
| The intervention prompts had a positive impact on my mental health | 0.01 [-0.02, 0.04] | 0.51 | |
| The intervention prompts got me to take action | 0.02 [-0.02, 0.05] | 0.30 | |
| Caregiver Strain | -0.00 [-0.00, 0.00] | 0.15 | |
| Caregiver Anxiety | -0.00 [-0.00, 0.00] | 0.61 | |
| Anger | -0.00 [-0.00, 0.00] | 0.21 | |
| Anxiety | -0.00 [-0.00, 0.00] | 0.16 | |
| Depression | -0.00 [-0.00, 0.00] | 0.17 | |
| Fatigue | -0.00 [-0.00, 0.00] | 0.37 | |
| Perceived stress | -0.00 [-0.00, 0.00] | 0.11 | |
| Positive affect | 0.00 [-0.00, 0.00] | 0.18 | |
| Ability to participate in social roles and activities | 0.00 [-0.00, 0.00] | 0.24 | |
| Sleep impairment | -0.00 [-0.00, 0.00] | 0.08 | |
| Global health - physical | -0.00 [-0.00, 0.00] | 0.41 | |
| Global health - mental | 0.00 [-0.00, 0.00] | 0.16 | |
| Steps | | |  |
| Age (care partner) | 0.00 [-0.00, 0.00] | 0.06 | |
| Age of person cared for | 0.00 [-0.00, 0.00] | 0.17 | |
| Sex (care partner) | 0.02 [-0.05, 0.08] | 0.63 | |
| Sex of person cared for | 0.06 [-0.00, 0.12] | 0.05 | |
| Race (care partner) |  | 0.04 | |
| Black vs. White | -0.09 [-0.17, -0.02] | 0.02 | |
| Other vs. White | -0.04 [-0.13, 0.05] | 0.37 | |
| Ethnicity (care partner) | 0.03 [-0.04, 0.10] | 0.44 | |
| Length of caregiving | 0.00 [-0.00, 0.01] | 0.10 | |
| Relation to care recipient |  | 0.23 | |
| Parent vs. Other | 0.03 [-0.04, 0.10] | 0.36 | |
| Partner vs. Other | 0.06 [-0.01, 0.12] | 0.09 | |
| Same household as care recipient (Yes vs. No) | -0.00 [-0.06, 0.06] | 0.95 | |
| Relationships status (care partner) (Married vs. Not Married) | 0.05 [-0.01, 0.12] | 0.08 | |
| Work status (care partner) (Full-time vs. Other Categories) | 0.03 [-0.02, 0.08] | 0.26 | |
| Eligibility rating (care partner) | -0.01 [-0.02, 0.00] | 0.11 | |
| Supervision rating scale | -0.00 [-0.01, 0.00] | 0.38 | |
| PCL5 | -0.00 [-0.00, 0.00] | 0.53 | |
| Time spent caregiving (≥4 hours vs. < 4 hours) | -0.07 [-0.13, -0.02] | 0.008 | |
| MPAI4 | 0.00 [-0.00, 0.00] | 0.98 | |
| The instructions for the Fitbit^®^ set-up were easy to understand | 0.05 [0.02, 0.08] | 0.0005 | |
| The Fitbit^®^ was easy to set up | 0.06 [0.03, 0.08] | 0.0001 | |
| The Fitbit^®^ was easy to use | 0.07 [0.04, 0.10] | <.0001 | |
| The Fitbit^®^ was comfortable to wear | 0.04 [0.01, 0.06] | 0.004 | |
| The Fitbit^®^ data was easy to sync with my phone | 0.06 [0.04, 0.08] | <.0001 | |
| I was confident using the Fitbit^®^ | 0.09 [0.06, 0.12] | <.0001 | |
| The instructions for the CareQOL app set-up were easy to understand | 0.01 [-0.02, 0.04] | 0.60 | |
| The CareQOL app was easy to set up | -0.00 [-0.04, 0.03] | 0.96 | |
| The CareQOL app was easy to use | 0.02 [-0.02, 0.06] | 0.25 | |
| I was confident using the CareQOL app | 0.02 [-0.02, 0.05] | 0.36 | |
| I am confident that I was using the CareQOL app correctly | 0.02 [-0.02, 0.06] | 0.35 | |
| What is your overall rating of the design of the screens on the app, including the colors and the layout | -0.01 [-0.03, 0.02] | 0.67 | |
| The daily questions on the app were easy to understand | -0.00 [-0.05, 0.04] | 0.86 | |
| The daily questions on the app were easy to answer | -0.00 [-0.04, 0.04] | 0.99 | |
| Answering the daily questions fit easily into my routine | 0.01 [-0.02, 0.05] | 0.47 | |
| The number of daily questions to answer on the app was reasonable | 0.03 [-0.02, 0.07] | 0.22 | |
| Did you receive assistance answering any of the daily questions on the app? | -0.01 [-0.11, 0.09] | 0.85 | |
| The monthly surveys on the app were easy to understand | -0.02 [-0.06, 0.03] | 0.44 | |
| The monthly surveys on the app were easy to answer | 0.00 [-0.03, 0.04] | 0.89 | |
| Answering the monthly surveys fit easily into my routine | -0.01 [-0.04, 0.02] | 0.57 | |
| The number of questions in the monthly surveys was reasonable | -0.00 [-0.03, 0.02] | 0.74 | |
| How likely are you to recommend this study to another family caregiver? | 0.01 [-0.02, 0.04] | 0.52 | |
| How likely are you to recommend the CareQOL app to another family caregiver? | 0.01 [-0.02, 0.04] | 0.46 | |
| The intervention prompts had a positive impact on my health | 0.02 [-0.02, 0.06] | 0.30 | |
| The intervention prompts had a positive impact on my mental health | 0.02 [-0.02, 0.05] | 0.41 | |
| The intervention prompts had a positive impact on my physical health | -0.01 [-0.04, 0.02] | 0.71 | |
| How often did you follow the recommendations made by the intervention prompts (e.g., focus on the positive, engage in activity, reach out for support) | 0.01 [-0.03, 0.04] | 0.79 | |
| The intervention prompts got me to take action | 0.00 [-0.04, 0.04] | 0.90 | |
| Caregiver Strain | 0.00 [-0.00, 0.01] | 0.13 | |
| Caregiver Anxiety | 0.00 [-0.00, 0.01] | 0.13 | |
| Anger | 0.00 [-0.00, 0.00] | 0.21 | |
| Anxiety | 0.00 [-0.00, 0.01] | 0.21 | |
| Depression | 0.00 [-0.00, 0.01] | 0.17 | |
| Fatigue | 0.00 [-0.00, 0.00] | 0.65 | |
| Perceived stress | 0.00 [-0.00, 0.00] | 0.92 | |
| Positive affect | -0.00 [-0.01, 0.00] | 0.49 | |
| Ability to participate in social roles and activities | -0.00 [-0.00, 0.00] | 0.86 | |
| Sleep impairment | 0.00 [-0.00, 0.00] | 0.99 | |
| Global health - physical | -0.00 [-0.01, 0.00] | 0.31 | |
| Global health - mental | -0.00 [-0.00, 0.00] | 0.37 | |
| Sleep | | |  |
| Age (care partner) | 0.00 [-0.00, 0.00] | 0.19 | |
| Age of person cared for | 0.00 [-0.00, 0.00] | 0.07 | |
| Sex (care partner) | 0.04 [-0.06, 0.14] | 0.46 | |
| Sex of person cared for | 0.09 [-0.01, 0.18] | 0.07 | |
| Race (care partner) |  | <.0001 | |
| Black vs. White | -0.31 [-0.42, -0.20] | <.0001 | |
| Other vs. White | -0.09 [-0.23, 0.05] | 0.20 | |
| Ethnicity (care partner) | 0.09 [-0.02, 0.20] | 0.13 | |
| Length of caregiving | 0.00 [-0.00, 0.01] | 0.48 | |
| Relation to care recipient |  | 0.16 | |
| Parent vs. Other | 0.05 [-0.06, 0.15] | 0.40 | |
| Partner vs. Other | 0.10 [-0.00, 0.20] | 0.06 | |
| Same household as care recipient (Yes vs. No) | 0.02 [-0.07, 0.12] | 0.61 | |
| Relationships status (care partner) (Married vs. Not Married) | 0.18 [0.09, 0.27] | 0.0002 | |
| Work status (care partner) (Full-time vs. Other Categories) | -0.02 [-0.10, 0.06] | 0.65 | |
| Eligibility rating (care partner) | -0.02 [-0.03, -0.00] | 0.04 | |
| Supervision rating scale | -0.00 [-0.01, 0.01] | 0.99 | |
| PCL5 | -0.00 [-0.00, 0.00] | 0.14 | |
| Time spent caregiving (≥4 hours vs. < 4 hours) | -0.09 [-0.17, 0.00] | 0.05 | |
| MPAI4 | 0.00 [-0.00, 0.00] | 0.96 | |
| The instructions for the Fitbit^®^ set-up were easy to understand | 0.03 [-0.02, 0.08] | 0.29 | |
| The Fitbit^®^ was easy to set up | 0.03 [-0.02, 0.08] | 0.25 | |
| The Fitbit^®^ was easy to use | 0.07 [0.02, 0.13] | 0.007 | |
| The Fitbit^®^ was comfortable to wear | 0.02 [-0.02, 0.07] | 0.31 | |
| The Fitbit^®^ data was easy to sync with my phone | 0.07 [0.03, 0.11] | 0.0007 | |
| I was confident using the Fitbit^®^ | 0.09 [0.04, 0.14] | 0.0006 | |
| The instructions for the CareQOL app set-up were easy to understand | 0.02 [-0.04, 0.08] | 0.49 | |
| The CareQOL app was easy to set up | -0.00 [-0.06, 0.06] | 0.91 | |
| The CareQOL app was easy to use | 0.02 [-0.05, 0.08] | 0.59 | |
| I was confident using the CareQOL app | 0.01 [-0.05, 0.07] | 0.83 | |
| I am confident that I was using the CareQOL app correctly | 0.03 [-0.03, 0.10] | 0.33 | |
| What is your overall rating of the design of the screens on the app, including the colors and the layout | -0.01 [-0.06, 0.03] | 0.57 | |
| The daily questions on the app were easy to understand | -0.02 [-0.10, 0.05] | 0.56 | |
| The daily questions on the app were easy to answer | -0.04 [-0.10, 0.02] | 0.24 | |
| Answering the daily questions fit easily into my routine | 0.00 [-0.06, 0.06] | 0.93 | |
| The number of daily questions to answer on the app was reasonable | 0.05 [-0.03, 0.12] | 0.21 | |
| Did you receive assistance answering any of the daily questions on the app? | -0.01 [-0.18, 0.16] | 0.92 | |
| The monthly surveys on the app were easy to understand | -0.04 [-0.11, 0.03] | 0.30 | |
| The monthly surveys on the app were easy to answer | -0.03 [-0.09, 0.03] | 0.35 | |
| Answering the monthly surveys fit easily into my routine | -0.02 [-0.08, 0.03] | 0.40 | |
| The number of questions in the monthly surveys was reasonable | 0.01 [-0.03, 0.05] | 0.67 | |
| How likely are you to recommend this study to another family caregiver? | 0.03 [-0.03, 0.08] | 0.32 | |
| How likely are you to recommend the CareQOL app to another family caregiver? | 0.02 [-0.03, 0.07] | 0.42 | |
| The intervention prompts had a positive impact on my health | 0.06 [0.00, 0.11] | 0.049 | |
| The intervention prompts had a positive impact on my mental health | 0.02 [-0.04, 0.08] | 0.47 | |
| The intervention prompts had a positive impact on my physical health | 0.02 [-0.03, 0.06] | 0.52 | |
| How often did you follow the recommendations made by the intervention prompts (e.g., focus on the positive, engage in activity, reach out for support) | 0.02 [-0.04, 0.08] | 0.50 | |
| The intervention prompts got me to take action | 0.03 [-0.04, 0.09] | 0.41 | |
| Caregiver Strain | 0.00 [-0.00, 0.01] | 0.18 | |
| Caregiver Anxiety | 0.00 [-0.00, 0.01] | 0.49 | |
| Anger | 0.00 [-0.00, 0.01] | 0.16 | |
| Anxiety | 0.00 [-0.00, 0.01] | 0.08 | |
| Depression | 0.00 [-0.00, 0.01] | 0.59 | |
| Fatigue | 0.00 [-0.00, 0.01] | 0.17 | |
| Perceived stress | 0.00 [-0.00, 0.00] | 0.71 | |
| Positive affect | -0.01 [-0.01, 0.00] | 0.10 | |
| Ability to participate in social roles and activities | -0.00 [-0.01, -0.00] | 0.048 | |
| Sleep impairment | 0.00 [-0.00, 0.00] | 0.71 | |
| Global health - physical | -0.00 [-0.01, 0.00] | 0.52 | |
| Global health - mental | -0.00 [-0.01, 0.00] | 0.09 | |
