# Supplementary material for: Completion and Compliance Rates for an Intensive mHealth Study Design to Promote Self-Awareness and Self-Care Among Care Partners of Individuals With Traumatic Brain Injury: Secondary Analysis of a Randomized Controlled Trial
Source: JMIR Mhealth Uhealth. 2025 Aug 21;13:e73772. doi: 10.2196/73772 (PMC12370270; doi:10.2196/73772)
Supplement: Multimedia Appendix 2 [file mhealth-v13-e73772-s002.docx]

|  | 2 cluster model | 3 cluster model | 4 cluster model |
| --- | --- | --- | --- |
| Within-cluster sum of squares | 0.1448 | 0.1169 | *0.1132* |
| Minimum distance between initial Seeds | *1.62584* | 1.025763 | 0.837552 |
| Pseudo F Statistic | *445.78* | 407.89 | 294.42 |
| Approximate R-Squared | 0.46627 | 0.61288 | *0.68319* |
| Cubic Clustering Criterion | 10.969 | *14.252* | 11.139 |

*Note.* italics indicate cluster with best model fit for a given approach
