# Supplementary material for: Completion and Compliance Rates for an Intensive mHealth Study Design to Promote Self-Awareness and Self-Care Among Care Partners of Individuals With Traumatic Brain Injury: Secondary Analysis of a Randomized Controlled Trial
Source: JMIR Mhealth Uhealth. 2025 Aug 21;13:e73772. doi: 10.2196/73772 (PMC12370270; doi:10.2196/73772)
Supplement: Checklist 1 [file mhealth-v13-e73772-s003.pdf]

CONSORT-EHEALTH (V 1.6.1) - Submission/Publication Form

The CONSORT-EHEALTH checklist is intended for authors of randomized trials evaluating web-based and Internet-based applications/interventions, including mobile interventions, electronic games (incl multiplayer games), social media, certain telehealth applications, and other interactive and/or networked electronic applications. Some of the items (e.g. all subitems under item 5 - description of the intervention) may also be applicable for other study designs.

The goal of the CONSORT EHEALTH checklist and guideline is to be
a) a guide for reporting for authors of RCTs,
b) to form a basis for appraisal of an ehealth trial (in terms of validity)

CONSORT-EHEALTH items/subitems are MANDATORY reporting items for studies published in the Journal of Medical Internet Research and other journals / scientific societies endorsing the checklist.

Items numbered 1., 2., 3., 4a., 4b etc are original CONSORT or CONSORT-NPT (non-pharmacologic treatment) items.
Items with Roman numerals (i., ii, iii, iv etc.) are CONSORT-EHEALTH extensions/clarifications.

As the CONSORT-EHEALTH checklist is still considered in a formative stage, we would ask that you also RATE ON A SCALE OF 1-5 how important/useful you feel each item is FOR THE PURPOSE OF THE CHECKLIST and reporting guideline (optional).

Mandatory reporting items are marked with a red *.
In the textboxes, either copy & paste the relevant sections from your manuscript into this form - please include any quotes from your manuscript in QUOTATION MARKS,
or answer directly by providing additional information not in the manuscript, or elaborating on why the item was not relevant for this study.

YOUR ANSWERS WILL BE PUBLISHED AS A SUPPLEMENTARY FILE TO YOUR PUBLICATION IN JMIR AND ARE CONSIDERED PART OF YOUR PUBLICATION (IF ACCEPTED).
Please fill in these questions diligently. Information will not be copyedited, so please use proper spelling and grammar, use correct capitalization, and avoid abbreviations.

DO NOT FORGET TO SAVE AS PDF _AND_ CLICK THE SUBMIT BUTTON SO YOUR ANSWERS ARE IN OUR DATABASE !!!

Citation Suggestion (if you append the pdf as Appendix we suggest to cite this paper in the caption):
Eysenbach G, CONSORT-EHEALTH Group

CONSORT-EHEALTH: Improving and Standardizing Evaluation Reports of Web-based and Mobile Health Interventions
J Med Internet Res 2011;13(4):e126
URL: http://www.jmir.org/2011/4/e126/
doi: 10.2196/jmir.1923
PMID: 22209829

Sign in to Google to save your progress. Learn more

* Indicates required question

Your name *
First Last

Noelle E Carlozzi

Primary Affiliation (short), City, Country *
University of Toronto, Toronto, Canada

University of Michigan

Your e-mail address *
abc@gmail.com

carlozzi@med.umich.edu

Title of your manuscript *
Provide the (draft) title of your manuscript.

Completion and compliance rates for an intensive mhealth study design to promote self-awareness and self-care among care partners of individuals with traumatic brain injury

Name of your App/Software/Intervention *

If there is a short and a long/alternate name, write the short name first and add the long name in brackets.

CareQOL App

Evaluated Version (if any)

e.g. "V1", "Release 2017-03-01", "Version 2.0.27913"

N/A

Language(s) *

What language is the intervention/app in? If multiple languages are available, separate by comma (e.g. "English, French")

English

URL of your Intervention Website or App

e.g. a direct link to the mobile app on app in appstore (itunes, Google Play), or URL of the website. If the intervention is a DVD or hardware, you can also link to an Amazon page.

No longer available

URL of an image/screenshot (optional)

N/A

Accessibility *

Can an enduser access the intervention presently?

- ○ access is free and open
- ○ access only for special usergroups, not open
- ○ access is open to everyone, but requires payment/subscription/in-app purchases
- ◉ app/intervention no longer accessible
- ○ Other:

Primary Medical Indication/Disease/Condition *

e.g. "Stress", "Diabetes", or define the target group in brackets after the condition, e.g. "Autism (Parents of children with)", "Alzheimers (Informal Caregivers of)"

Traumatic Brain Injury (Informal Caregivers of)

Primary Outcomes measured in trial *

comma-separated list of primary outcomes reported in the trial

Primary outcomes are reported in Carlozzi et al., 2025,Improving Outcomes for Care Partners of Individuals With Traumatic Brain Injury: Results for a mHealth Randomized Control Trial of the CareQOL App, Archives of Physical Medicine and Rehabilitation

Secondary/other outcomes

Are there any other outcomes the intervention is expected to affect?

Secondary outcomes are reported in Carlozzi et al., 2025,Improving Outcomes for Care Partners of Individuals With Traumatic Brain Injury: Results for a mHealth Randomized Control Trial of the CareQOL App, Archives of Physical Medicine and Rehabilitation

Recommended "Dose" *

What do the instructions for users say on how often the app should be used?

- (selected) Approximately Daily
- Approximately Weekly
- Approximately Monthly
- Approximately Yearly
- "as needed"
- Other:

Approx. Percentage of Users (starters) still using the app as recommended after 3 months *

- unknown / not evaluated
- 0-10%
- 11-20%
- 21-30%
- 31-40%
- 41-50%
- 51-60%
- 61-70%
- 71%-80%
- 81-90%
- (selected) 91-100%
- Other:

Overall, was the app/intervention effective? *

- ○ yes: all primary outcomes were significantly better in intervention group vs control
- ○ partly: SOME primary outcomes were significantly better in intervention group vs control
- ○ no statistically significant difference between control and intervention
- ○ potentially harmful: control was significantly better than intervention in one or more outcomes
- ○ inconclusive: more research is needed
- ◉ Other: Although we did not see improvements in HRQOL outcomes, nor in ph

Article Preparation Status/Stage *

At which stage in your article preparation are you currently (at the time you fill in this form)

- ○ not submitted yet - in early draft status
- ○ not submitted yet - in late draft status, just before submission
- ○ submitted to a journal but not reviewed yet
- X submitted to a journal and after receiving initial reviewer comments
- ○ submitted to a journal and accepted, but not published yet
- ○ published
- ○ Other:

Journal *

If you already know where you will submit this paper (or if it is already submitted), please provide the journal name (if it is not JMIR, provide the journal name under "other")

- ○ not submitted yet / unclear where I will submit this
- ◉ Journal of Medical Internet Research (JMIR)
- ○ JMIR mHealth and UHealth
- ○ JMIR Serious Games
- ○ JMIR Mental Health
- ○ JMIR Public Health
- ○ JMIR Formative Research
- ○ Other JMIR sister journal
- ○ Other:

Is this a full powered effectiveness trial or a pilot/feasibility trial? *

- ○ Pilot/feasibility
- ◉ Fully powered

Manuscript tracking number *

If this is a JMIR submission, please provide the manuscript tracking number under "other" (The ms tracking number can be found in the submission acknowledgement email, or when you login as author in JMIR. If the paper is already published in JMIR, then the ms tracking number is the four-digit number at the end of the DOI, to be found at the bottom of each published article in JMIR)

- ○ no ms number (yet) / not (yet) submitted to / published in JMIR
- ◉ Other: #73772

TITLE AND ABSTRACT

1a) TITLE: Identification as a randomized trial in the title

1a) Does your paper address CONSORT item 1a? *

I.e does the title contain the phrase "Randomized Controlled Trial"? (if not, explain the reason under "other")

- ○ yes
- X Other: Given that we are examining completion and compliance rates, the fact that the completion/compliance rates are from an RCT is not especially n oteworthy

1a-i) Identify the mode of delivery in the title

Identify the mode of delivery. Preferably use “web-based” and/or “mobile” and/or “electronic game” in the title. Avoid ambiguous terms like “online”, “virtual”, “interactive”. Use “Internet-based” only if Intervention includes non-web-based Internet components (e.g. email), use “computer-based” or “electronic” only if offline products are used. Use “virtual” only in the context of “virtual reality” (3-D worlds). Use “online” only in the context of “online support groups”. Complement or substitute product names with broader terms for the class of products (such as “mobile” or “smart phone” instead of “iphone”), especially if the application runs on different platforms.

	1	2	3	4	5	
subitem not at all important	○	●	○	○	○	essential

Clear selection

Does your paper address subitem 1a-i? *

Copy and paste relevant sections from manuscript title (include quotes in quotation marks "like this" to indicate direct quotes from your manuscript), or elaborate on this item by providing additional information not in the ms, or briefly explain why the item is not applicable/relevant for your study

This is a secondary analysis that explores the completion/compliance rates in this RCT, as such, subitem 1A-i is not especially relevant; we do indicate that this is a mHealth study in the title

1a-ii) Non-web-based components or important co-interventions in title

Mention non-web-based components or important co-interventions in title, if any (e.g., “with telephone support”).

	1	2	3	4	5	
subitem not at all important	●	○	○	○	○	essential

Clear selection

Does your paper address subitem 1a-ii?

Copy and paste relevant sections from manuscript title (include quotes in quotation marks "like this" to indicate direct quotes from your manuscript), or elaborate on this item by providing additional information not in the ms, or briefly explain why the item is not applicable/relevant for your study

This is a secondary analysis that explores the completion/compliance rates in this RCT, as such, subitem 1A-ii is not especially relevant

1a-iii) Primary condition or target group in the title

Mention primary condition or target group in the title, if any (e.g., “for children with Type I Diabetes”) Example: A Web-based and Mobile Intervention with Telephone Support for Children with Type I Diabetes: Randomized Controlled Trial

	1	2	3	4	5	
subitem not at all important	○	○	○	○	●	essential

Clear selection

Does your paper address subitem 1a-iii? *

Copy and paste relevant sections from manuscript title (include quotes in quotation marks "like this" to indicate direct quotes from your manuscript), or elaborate on this item by providing additional information not in the ms, or briefly explain why the item is not applicable/relevant for your study

yes: care partners of individuals with traumatic brain injury

1b) ABSTRACT: Structured summary of trial design, methods, results, and conclusions

NPT extension: Description of experimental treatment, comparator, care providers, centers, and blinding status.

1b-i) Key features/functionalities/components of the intervention and comparator in the METHODS section of the ABSTRACT

Mention key features/functionalities/components of the intervention and comparator in the abstract. If possible, also mention theories and principles used for designing the site. Keep in mind the needs of systematic reviewers and indexers by including important synonyms. (Note: Only report in the abstract what the main paper is reporting. If this information is missing from the main body of text, consider adding it)

	1	2	3	4	5	
subitem not at all important	○	○ x	○	○	○	essential

Clear selection

Does your paper address subitem 1b-i? *

Copy and paste relevant sections from the manuscript abstract (include quotes in quotation marks "like this" to indicate direct quotes from your manuscript), or elaborate on this item by providing additional information not in the ms, or briefly explain why the item is not applicable/relevant for your study

"This randomized controlled trial was designed to test the CareQOL app, a mobile health app designed to promote care partner self-awareness (through self-monitoring) and self-care (through personalized self-care push notifications). "

1b-ii) Level of human involvement in the METHODS section of the ABSTRACT

Clarify the level of human involvement in the abstract, e.g., use phrases like "fully automated" vs. "therapist/nurse/care provider/physician-assisted" (mention number and expertise of providers involved, if any). (Note: Only report in the abstract what the main paper is reporting. If this information is missing from the main body of text, consider adding it)

	1	2	3	4	5	
subitem not at all important	○	○	○	○	◉	essential

Clear selection

Does your paper address subitem 1b-ii?

Copy and paste relevant sections from the manuscript abstract (include quotes in quotation marks "like this" to indicate direct quotes from your manuscript), or elaborate on this item by providing additional information not in the ms, or briefly explain why the item is not applicable/relevant for your study

The study design consisted of 1) a baseline assessment of several care partner patient-reported outcome measures (PROs) that assessed health-related quality of life (HRQOL) and proxy measures of the functional/mental status of the person with TBI; 2) a 6-month home monitoring period that included three daily ecological momentary assessment (EMA) questions, monthly PRO surveys assessing 12 HRQOL domains, and continuous activity and sleep monitoring using a Fitbit®; and 3) two follow-up PRO surveys assessing care partner HRQOL at 3 and 6 months post-home monitoring.

1b-iii) Open vs. closed, web-based (self-assessment) vs. face-to-face assessments in the METHODS section of the ABSTRACT

Mention how participants were recruited (online vs. offline), e.g., from an open access website or from a clinic or a closed online user group (closed usergroup trial), and clarify if this was a purely web-based trial, or there were face-to-face components (as part of the intervention or for assessment). Clearly say if outcomes were self-assessed through questionnaires (as common in web-based trials). Note: In traditional offline trials, an open trial (open-label trial) is a type of clinical trial in which both the researchers and participants know which treatment is being administered. To avoid confusion, use “blinded” or “unblinded” to indicated the level of blinding instead of “open”, as “open” in web-based trials usually refers to “open access” (i.e. participants can self-enrol). (Note: Only report in the abstract what the main paper is reporting. If this information is missing from the main body of text, consider adding it)

	1	2	3	4	5	
subitem not at all important	○	◉	○	○	○	essential

Clear selection

Does your paper address subitem 1b-iii?

Copy and paste relevant sections from the manuscript abstract (include quotes in quotation marks "like this" to indicate direct quotes from your manuscript), or elaborate on this item by providing additional information not in the ms, or briefly explain why the item is not applicable/relevant for your study

Given that this is a secondary analysis, this is described in the methods section: Participants were recruited through two academic medical centers using clinical databases,24 site-specific registries, and community outreach. Recruitment often included contacting a person with known TBI for their care partner referrals. Care partners needed to be at least 18 years of age, able to read and understand English, and caring for an adult at least one-year post-injury who had sustained a medically documented complicated mild, moderate, or severe TBI. The injury must have occurred when the care recipient was age 16 or older. A care partner was defined as an individual who provided assistance to a person with a TBI (indicated by a response greater than 0 on the following question: “On a scale of 0-10, where 0 is ‘no assistance’ and 10 is ‘assistance with all activities,’ how much assistance does the person you care for require from you to complete activities of daily living due to problems resulting from his/her TBI?”). Care partners were excluded if they did not have access to resources for participating in a mHealth intervention, including a personal mobile device capable of downloading the study apps for this study. Participants had to be willing to download the CareQOL and Fitbit® apps to their device and be willing to complete all study assessments. In addition, we excluded professional, paid caregivers.

1b-iv) RESULTS section in abstract must contain use data

Report number of participants enrolled/assessed in each group, the use/uptake of the intervention (e.g., attrition/adherence metrics, use over time, number of logins etc.), in addition to primary/secondary outcomes. (Note: Only report in the abstract what the main paper is reporting. If this information is missing from the main body of text, consider adding it)

	1	2	3	4	5	
subitem not at all important	○	○	○	○	◉	essential

Clear selection

Does your paper address subitem 1b-iv?

Copy and paste relevant sections from the manuscript abstract (include quotes in quotation marks "like this" to indicate direct quotes from your manuscript), or elaborate on this item by providing additional information not in the ms, or briefly explain why the item is not applicable/relevant for your study

Overall compliance for the different aspects of the study was high. On average, the full-sample daily EMA PROs completion rate was 84%, Fitbit®-based step count compliance was 90%, and Fitbit®-based sleep duration compliance was 75%; there was no difference between the study arms for daily EMA PROs and Fitbit® compliance rates. Completion rates for monthly and follow-up PRO surveys were even higher, with average end-of-month completion rates ranging from 94% to 98%, and follow-up completion rates of 92% for both time points. Again, these rates did not differ by study arm.

1b-v) CONCLUSIONS/DISCUSSION in abstract for negative trials

Conclusions/Discussions in abstract for negative trials: Discuss the primary outcome - if the trial is negative (primary outcome not changed), and the intervention was not used, discuss whether negative results are attributable to lack of uptake and discuss reasons. (Note: Only report in the abstract what the main paper is reporting. If this information is missing from the main body of text, consider adding it)

	1	2	3	4	5	
subitem not at all important	◉	○	○	○	○	essential

Clear selection

Does your paper address subitem 1b-v?

Copy and paste relevant sections from the manuscript abstract (include quotes in quotation marks "like this" to indicate direct quotes from your manuscript), or elaborate on this item by providing additional information not in the ms, or briefly explain why the item is not applicable/relevant for your study

This is a secondary data analysis focused on completion rates and compliance, as such, this is what the discussion focuses on: The compliance rates for this intensive study design are consistent, but at the high end, with what has been reported previously in the literature for studies with shorter time durations. We anticipate the high compliance rates observed in the current study likely are due to several study-specific design elements that were employed to encourage study engagement.

INTRODUCTION

2a) In INTRODUCTION: Scientific background and explanation of rationale

2a-i) Problem and the type of system/solution

Describe the problem and the type of system/solution that is object of the study: intended as stand-alone intervention vs. incorporated in broader health care program? Intended for a particular patient population? Goals of the intervention, e.g., being more cost-effective to other interventions, replace or complement other solutions? (Note: Details about the intervention are provided in “Methods” under 5)

	1	2	3	4	5	
subitem not at all important	○	○	○	○	●	essential

Clear selection

Does your paper address subitem 2a-i? *

Copy and paste relevant sections from the manuscript (include quotes in quotation marks "like this" to indicate direct quotes from your manuscript), or elaborate on this item by providing additional information not in the ms, or briefly explain why the item is not applicable/relevant for your study

Compliance rates for mobile health (mHealth) studies that involve ecological momentary assessments (EMAs) are highly variable. In general, compliance for brief, intensive mHealth studies (i.e., 2 weeks or less) is moderately high, ranging from 57% to 87%;1-7 these rates tend to progressively worsen as the study duration increases (ranging from 55% to 90% for studies lasting two to four weeks8-17 and 77% for the single study we found that included a study duration of greater than 3 months18). These high rates of variability are due to both person-specific (e.g., personality, comfort with technology and wearable devices19) and protocol-specific factors (e.g., total number of questions, study duration, compensation rates, criteria for compensation20) (see Table 1).

In our own work, we have demonstrated compliance rates at the high end of the above ranges. Specifically, in a one-week study that included 3 daily EMA questions, we found an average response rate of 83%. In another, longer study (3-months duration), we found an average response rate of 90% for once-daily EMA questions, and 96% and 85% for daily step count data and sleep duration estimates, respectively (derived from continuous monitoring with a wearable device).21 We believe that these high compliance rates can be attributed, at least in part, to specific study design elements, including monetary compensation for data contribution ($1 per day for either EMA or device data, and a graduated compensation plan for each of the monthly and monthly follow-up surveys of $10 for each monthly survey completed in months 1-5, and $20 for completing the final (month 6) survey. Additionally, participants were paid $20 for the completion of the two post-program surveys and were allowed to keep the study-provided wearable device after the completion of their participation in the study. The brevity of overall assessments, customization of administration windows for the EMA questions, and regular reminders following a 3-day lapse in responding also may have contributed to the higher response rates.22

Care partners of individuals with traumatic brain injury (TBI) are a population that may potentially have difficulty engaging with EMA over longer time periods, due to caregiving demands and/or stress related to the caregiving role. We engaged a sample of care partners of individuals with TBI in an intensive study design that was of a longer duration (i.e., a 12-month study that included 6 months of EMA PROs).23 In this study, participants completed a once-daily, EMAs (3 items), wore a Fitbit®, completed end-of-month surveys for 6 months, and completed follow-up surveys at 9 and 12 months. Previous examination of this dataset indicated high rates of compliance, with average completion rates of 84% (SD=19) for once-daily EMA questions, and 90% (SD=21) and 75% (SD=32) for daily step count and sleep duration estimates, respectively (derived from continuous monitoring with a Fitbit®).23 For the purposes of this analysis, we wished to explore these rates more closely and determine what, if any, additional demographic factors were related to compliance rates in our sample.

Also see Table 1.

2a-ii) Scientific background, rationale: What is known about the (type of) system

Scientific background, rationale: What is known about the (type of) system that is the object of the study (be sure to discuss the use of similar systems for other conditions/diagnoses, if appropiate), motivation for the study, i.e. what are the reasons for and what is the context for this specific study, from which stakeholder viewpoint is the study performed, potential impact of findings [2]. Briefly justify the choice of the comparator.

	1	2	3	4	5	
subitem not at all important	○	○	○	○	◉	essential

Clear selection

Does your paper address subitem 2a-ii? *

Copy and paste relevant sections from the manuscript (include quotes in quotation marks "like this" to indicate direct quotes from your manuscript), or elaborate on this item by providing additional information not in the ms, or briefly explain why the item is not applicable/relevant for your study

Compliance rates for mobile health (mHealth) studies that involve ecological momentary assessments (EMAs) are highly variable. In general, compliance for brief, intensive mHealth studies (i.e., 2 weeks or less) is moderately high, ranging from 57% to 87%;1-7 these rates tend to progressively worsen as the study duration increases (ranging from 55% to 90% for studies lasting two to four weeks8-17 and 77% for the single study we found that included a study duration of greater than 3 months18). These high rates of variability are due to both person-specific (e.g., personality, comfort with technology and wearable devices19) and protocol-specific factors (e.g., total number of questions, study duration, compensation rates, criteria for compensation20) (see Table 1).

In our own work, we have demonstrated compliance rates at the high end of the above ranges. Specifically, in a one-week study that included 3 daily EMA questions, we found an average response rate of 83%. In another, longer study (3-months duration), we found an average response rate of 90% for once-daily EMA questions, and 96% and 85% for daily step count data and sleep duration estimates, respectively (derived from continuous monitoring with a wearable device).21 We believe that these high compliance rates can be attributed, at least in part, to specific study design elements, including monetary compensation for data contribution ($1 per day for either EMA or device data, and a graduated compensation plan for each of the monthly and monthly follow-up surveys of $10 for each monthly survey completed in months 1-5, and $20 for completing the final (month 6) survey. Additionally, participants were paid $20 for the completion of the two post-program surveys and were allowed to keep the study-provided wearable device after the completion of their participation in the study. The brevity of overall assessments, customization of administration windows for the EMA questions, and regular reminders following a 3-day lapse in responding also may have contributed to the higher response rates.22

Also see Table 1.

2b) In INTRODUCTION: Specific objectives or hypotheses

Does your paper address CONSORT subitem 2b? *

Copy and paste relevant sections from the manuscript (include quotes in quotation marks "like this" to indicate direct quotes from your manuscript), or elaborate on this item by providing additional information not in the ms, or briefly explain why the item is not applicable/relevant for your study

Care partners of individuals with traumatic brain injury (TBI) are a population that may potentially have difficulty engaging with EMA over longer time periods, due to caregiving demands and/or stress related to the caregiving role. We engaged a sample of care partners of individuals with TBI in an intensive study design that was of a longer duration (i.e., a 12-month study that included 6 months of EMA PROs).23 In this study, participants completed a once-daily, EMAs (3 items), wore a Fitbit®, completed end-of-month surveys for 6 months, and completed follow-up surveys at 9 and 12 months. Previous examination of this dataset indicated high rates of compliance, with average completion rates of 84% (SD=19) for once-daily EMA questions, and 90% (SD=21) and 75% (SD=32) for daily step count and sleep duration estimates, respectively (derived from continuous monitoring with a Fitbit®).23 For the purposes of this analysis, we wished to explore these rates more closely and determine what, if any, additional demographic factors were related to compliance rates in our sample.

METHODS

3a) Description of trial design (such as parallel, factorial) including allocation ratio

Does your paper address CONSORT subitem 3a? *

Copy and paste relevant sections from the manuscript (include quotes in quotation marks "like this" to indicate direct quotes from your manuscript), or elaborate on this item by providing additional information not in the ms, or briefly explain why the item is not applicable/relevant for your study

Participants were randomized to either a self-monitoring alone arm which included completion of the daily EMA questions, baseline, monthly, and follow-up PRO surveys, and 6 months of continuous activity and sleep monitoring with a Fitbit®, or to a self-monitoring plus self-care push notifications arm which included self-monitoring plus self-care push notifications which involved a 50/50 chance each day of receiving a self-care prompt in addition to the other assessments. All participants had access to a self-monitoring dashboard (CareQOL app) that included graphical displays of the daily EMA scores as well as daily step count and sleep duration data from the Fitbit®. Several electronic data capture and management platforms were employed, including REDCap, CareQOL, Qualtrics, Fitbit®, the University of Michigan Health Information Technology and Services server, and the Google Cloud.

More detail is provided in a previously published manuscripts:
Carlozzi, N.E., Troost, J.P., Sen, S., Choi, S.W., Wu, Z., Miner, J.A., Lombard, W.L., Graves, C., Sander, A.M. (In Press). Improving outcomes for care partners of individuals with traumatic brain injury: Results for a mHealth randomized control trial of the CareQOL app. Archives of Physical Medicine and Rehabilitation.

Carlozzi NE, Sander AM, Choi SW, et al. Improving outcomes for care partners of persons with traumatic brain injury: Protocol for a randomized control trial of a just-in-time-adaptive self-management intervention. PLoS One. 2022;17(6):e0268726.

3b) Important changes to methods after trial commencement (such as eligibility criteria), with reasons

Does your paper address CONSORT subitem 3b? *

Copy and paste relevant sections from the manuscript (include quotes in quotation marks "like this" to indicate direct quotes from your manuscript), or elaborate on this item by providing additional information not in the ms, or briefly explain why the item is not applicable/relevant for your study

previously published in Carlozzi, N.E., Troost, J.P., Sen, S., Choi, S.W., Wu, Z., Miner, J.A., Lombard, W.L., Graves, C., Sander, A.M. (In Press). Improving outcomes for care partners of individuals with traumatic brain injury: Results for a mHealth randomized control trial of the CareQOL app. Archives of Physical Medicine and Rehabilitation.

3b-i) Bug fixes, Downtimes, Content Changes

Bug fixes, Downtimes, Content Changes: ehealth systems are often dynamic systems. A description of changes to methods therefore also includes important changes made on the intervention or comparator during the trial (e.g., major bug fixes or changes in the functionality or content) (5-iii) and other “unexpected events” that may have influenced study design such as staff changes, system failures/downtimes, etc. [2].

	1	2	3	4	5	
subitem not at all important	(●)	( )	( )	( )	( )	essential

Clear selection

Does your paper address subitem 3b-i?

Copy and paste relevant sections from the manuscript (include quotes in quotation marks "like this" to indicate direct quotes from your manuscript), or elaborate on this item by providing additional information not in the ms, or briefly explain why the item is not applicable/relevant for your study

covered in previously published manuscript

4a) Eligibility criteria for participants

Does your paper address CONSORT subitem 4a? *

Copy and paste relevant sections from the manuscript (include quotes in quotation marks "like this" to indicate direct quotes from your manuscript), or elaborate on this item by providing additional information not in the ms, or briefly explain why the item is not applicable/relevant for your study

A total of 254 care partners of people with TBI participated in this study. Details describing the demographic data for the different study arms have been published previously (see Carlozzi et al.23). Table 2 provides an abbreviated summary of the demographic data for the full sample.

Overall compliance for the different aspects of the study was high (see Tables 3 & 4). Specifically, on average, the full-sample daily EMA completion rate was 84%, Fitbit®-based step count compliance was 90%, and Fitbit®-based sleep duration compliance was 75%; there was no difference between the study arms for daily completion and compliance rates (Table 3). Completion rates for monthly and follow-up surveys were even higher, with average end-of-month completion rates ranging from 94% to 98%, and follow-up completion rates of 92% for both 3-month and 6-month post time points; again, these rates did not differ by study arm (Table 4). Compliance rates were moderately correlated for the two Fitbit®-based measures ($r = 0.65$), and the magnitude of the correlations were less robust between Fitbit®-based compliance data and the daily EMAs ($r = 0.38$ between steps and EMAs and $r = 0.29$ between sleep and EMAs).

4a-i) Computer / Internet literacy

Computer / Internet literacy is often an implicit “de facto” eligibility criterion - this should be explicitly clarified.

	1	2	3	4	5	
subitem not at all important	◉	○	○	○	○	essential

Clear selection

Does your paper address subitem 4a-i?

Copy and paste relevant sections from the manuscript (include quotes in quotation marks "like this" to indicate direct quotes from your manuscript), or elaborate on this item by providing additional information not in the ms, or briefly explain why the item is not applicable/relevant for your study

Given that this is a secondary analysis, this information is more appropriate to the primary paper.

4a-ii) Open vs. closed, web-based vs. face-to-face assessments:

Open vs. closed, web-based vs. face-to-face assessments: Mention how participants were recruited (online vs. offline), e.g., from an open access website or from a clinic, and clarify if this was a purely web-based trial, or there were face-to-face components (as part of the intervention or for assessment), i.e., to what degree got the study team to know the participant. In online-only trials, clarify if participants were quasi-anonymous and whether having multiple identities was possible or whether technical or logistical measures (e.g., cookies, email confirmation, phone calls) were used to detect/prevent these.

	1	2	3	4	5	
subitem not at all important	○	○	◉	○	○	essential

Clear selection

Does your paper address subitem 4a-ii? *

Copy and paste relevant sections from the manuscript (include quotes in quotation marks "like this" to indicate direct quotes from your manuscript), or elaborate on this item by providing additional information not in the ms, or briefly explain why the item is not applicable/relevant for your study

Participants were recruited through two academic medical centers using clinical databases,24 site-specific registries, and community outreach. Recruitment often included contacting a person with known TBI for their care partner referrals.

More detail is reported in:
Carlozzi, N.E., Troost, J.P., Sen, S., Choi, S.W., Wu, Z., Miner, J.A., Lombard, W.L., Graves, C., Sander, A.M. (In Press). Improving outcomes for care partners of individuals with traumatic brain injury: Results for a mHealth randomized control trial of the CareQOL app. Archives of Physical Medicine and Rehabilitation.

Carlozzi NE, Sander AM, Choi SW, et al. Improving outcomes for care partners of persons with traumatic brain injury: Protocol for a randomized control trial of a just-in-time-adaptive self-management intervention. PLoS One. 2022;17(6):e0268726.

4a-iii) Information giving during recruitment

Information given during recruitment. Specify how participants were briefed for recruitment and in the informed consent procedures (e.g., publish the informed consent documentation as appendix, see also item X26), as this information may have an effect on user self-selection, user expectation and may also bias results.

	1	2	3	4	5	
subitem not at all important	(selected)	○	○	○	○	essential

Clear selection

Does your paper address subitem 4a-iii?

Copy and paste relevant sections from the manuscript (include quotes in quotation marks "like this" to indicate direct quotes from your manuscript), or elaborate on this item by providing additional information not in the ms, or briefly explain why the item is not applicable/relevant for your study

This was reported in Carlozzi, N.E., Troost, J.P., Sen, S., Choi, S.W., Wu, Z., Miner, J.A., Lombard, W.L., Graves, C., Sander, A.M. (In Press). Improving outcomes for care partners of individuals with traumatic brain injury: Results for a mHealth randomized control trial of the CareQOL app. Archives of Physical Medicine and Rehabilitation.

4b) Settings and locations where the data were collected

Does your paper address CONSORT subitem 4b? *

Copy and paste relevant sections from the manuscript (include quotes in quotation marks "like this" to indicate direct quotes from your manuscript), or elaborate on this item by providing additional information not in the ms, or briefly explain why the item is not applicable/relevant for your study

Previously reported in: Carlozzi, N.E., Troost, J.P., Sen, S., Choi, S.W., Wu, Z., Miner, J.A., Lombard, W.L., Graves, C., Sander, A.M. (In Press). Improving outcomes for care partners of individuals with traumatic brain injury: Results for a mHealth randomized control trial of the CareQOL app. Archives of Physical Medicine and Rehabilitation.

4b-i) Report if outcomes were (self-)assessed through online questionnaires

Clearly report if outcomes were (self-)assessed through online questionnaires (as common in web-based trials) or otherwise.

	1	2	3	4	5	
subitem not at all important	○	○	○	○	◉	essential

Clear selection

Does your paper address subitem 4b-i? *

Copy and paste relevant sections from the manuscript (include quotes in quotation marks "like this" to indicate direct quotes from your manuscript), or elaborate on this item by providing additional information not in the ms, or briefly explain why the item is not applicable/relevant for your study

A detailed description of the study protocol is provided elsewhere.25 Briefly, participants completed a baseline assessment assessing demographic variables and measures of the care recipient's functional and emotional status (Supervision Rating Scale [SRS],26 Mayo-Portland Adaptability Inventory-Fourth Edition [MPAI-4],27 and the Posttraumatic Stress Disorder Checklist for DSM-5 [PCL-5]28) and 12 HRQOL PROs (Caregiver Strain,29, 30 Caregiver-Specific Anxiety,30, 31 PROMIS Sleep-Related Impairment,32 PROMIS Fatigue,32 Anxiety,32 Depression,32 Anger,32 Self-Efficacy-General,33 Positive Affect and Well-Being,32 Perceived Stress,33 Ability to Participate in Social Roles and Activities,32 and Global Health34). This was followed by a 6-month home monitoring period that included 3 daily EMA questions (single-item assessments of Caregiver Strain,29, 30 PROMIS Anxiety32 and PROMIS Depression32), monthly surveys (again assessing the 12 HRQOL domains) as well as continuous monitoring of physical activity and sleep monitoring using a Fitbit.® The 3- and 6-month follow-up HRQOL PROs were identical to the end of month PROs. In addition, a feasibility and acceptability survey was also administered at the end of month 6.21

Study Procedures
Participants were randomized to either a self-monitoring alone arm which included completion of the daily EMA questions, baseline, monthly, and follow-up PRO surveys, and 6 months of continuous activity and sleep monitoring with a Fitbit®, or to a self-monitoring plus self-care push notifications arm which included self-monitoring plus self-care push notifications which involved a 50/50 chance each day of receiving a self-care prompt in addition to the other assessments. All participants had access to a self-monitoring dashboard (CareQOL app) that included graphical displays of the daily EMA scores as well as daily step count and sleep duration data from the Fitbit®. Several electronic data capture and management platforms were employed, including REDCap, CareQOL, Qualtrics, Fitbit®, the University of Michigan Health Information Technology and Services server, and the Google Cloud.

4b-ii) Report how institutional affiliations are displayed

Report how institutional affiliations are displayed to potential participants [on ehealth media], as affiliations with prestigious hospitals or universities may affect volunteer rates, use, and reactions with regards to an intervention.(Not a required item – describe only if this may bias results)

	1	2	3	4	5	
subitem not at all important	◉	○	○	○	○	essential

Clear selection

Does your paper address subitem 4b-ii?

Copy and paste relevant sections from the manuscript (include quotes in quotation marks "like this" to indicate direct quotes from your manuscript), or elaborate on this item by providing additional information not in the ms, or briefly explain why the item is not applicable/relevant for your study

Institutional information was not displayed

5) The interventions for each group with sufficient details to allow replication, including how and when they were actually administered

5-i) Mention names, credential, affiliations of the developers, sponsors, and owners

Mention names, credential, affiliations of the developers, sponsors, and owners [6] (if authors/evaluators are owners or developer of the software, this needs to be declared in a “Conflict of interest” section or mentioned elsewhere in the manuscript).

	1	2	3	4	5	
subitem not at all important	○	○	◉	○	○	essential

Clear selection

Does your paper address subitem 5-i?

Copy and paste relevant sections from the manuscript (include quotes in quotation marks "like this" to indicate direct quotes from your manuscript), or elaborate on this item by providing additional information not in the ms, or briefly explain why the item is not applicable/relevant for your study

Relevant Information was included in Acknowledgements:

Work on this manuscript was supported by grant numbers R01NR013658 from the National Institutes of Health (NIH), National Institute of Nursing Research, R01HL146354 and K24HL156896 from the National Heart, Lung and Blood Institute, and UL1TR002240 from the National Center for Advancing Translational Sciences. This work was also supported by the University of Michigan Institute for Healthcare Policy and Innovation. We thank the investigators, coordinators, and research associates/assistants who worked on this study, the study participants, and organizations who supported recruitment efforts.

Site Investigators and Coordinators: Noelle Carlozzi, Sung Won Choi, Zhenke Wu, Srijan Sen, Christopher Graves, Angela Lyden, Nikki Hubbard, Abigail Biddix, Jennifer Miner (University of Michigan, Ann, Arbor, MI); Angelle Sander (Baylor College of Medicine and TIRR Memorial Hermann, Houston, TX), Jay Bogaards (TIRR Memorial Hermann, Houston, TX)

Conflicts of Interest
The authors have no conflicts of interest to report regarding this publication.

5-ii) Describe the history/development process

Describe the history/development process of the application and previous formative evaluations (e.g., focus groups, usability testing), as these will have an impact on adoption/use rates and help with interpreting results.

	1	2	3	4	5	
subitem not at all important	(●)	( )	( )	( )	( )	essential

Clear selection

Does your paper address subitem 5-ii?

Copy and paste relevant sections from the manuscript (include quotes in quotation marks "like this" to indicate direct quotes from your manuscript), or elaborate on this item by providing additional information not in the ms, or briefly explain why the item is not applicable/relevant for your study

This was reported in Carlozzi, N.E., Troost, J.P., Sen, S., Choi, S.W., Wu, Z., Miner, J.A., Lombard, W.L., Graves, C., Sander, A.M. (In Press). Improving outcomes for care partners of individuals with traumatic brain injury: Results for a mHealth randomized control trial of the CareQOL app. Archives of Physical Medicine and Rehabilitation.

5-iii) Revisions and updating

Revisions and updating. Clearly mention the date and/or version number of the application/intervention (and comparator, if applicable) evaluated, or describe whether the intervention underwent major changes during the evaluation process, or whether the development and/or content was “frozen” during the trial. Describe dynamic components such as news feeds or changing content which may have an impact on the replicability of the intervention (for unexpected events see item 3b).

	1	2	3	4	5	
subitem not at all important	◉	○	○	○	○	essential

Clear selection

Does your paper address subitem 5-iii?

Copy and paste relevant sections from the manuscript (include quotes in quotation marks "like this" to indicate direct quotes from your manuscript), or elaborate on this item by providing additional information not in the ms, or briefly explain why the item is not applicable/relevant for your study

The same version was used throughout the study duration.

5-iv) Quality assurance methods

Provide information on quality assurance methods to ensure accuracy and quality of information provided [1], if applicable.

	1	2	3	4	5	
subitem not at all important	◉	○	○	○	○	essential

Clear selection

Does your paper address subitem 5-iv?

Copy and paste relevant sections from the manuscript (include quotes in quotation marks "like this" to indicate direct quotes from your manuscript), or elaborate on this item by providing additional information not in the ms, or briefly explain why the item is not applicable/relevant for your study

This was reported in Carlozzi, N.E., Troost, J.P., Sen, S., Choi, S.W., Wu, Z., Miner, J.A., Lombard, W.L., Graves, C., Sander, A.M. (In Press). Improving outcomes for care partners of individuals with traumatic brain injury: Results for a mHealth randomized control trial of the CareQOL app. Archives of Physical Medicine and Rehabilitation.

5-v) Ensure replicability by publishing the source code, and/or providing screenshots/screen-capture video, and/or providing flowcharts of the algorithms used

Ensure replicability by publishing the source code, and/or providing screenshots/screen-capture video, and/or providing flowcharts of the algorithms used. Replicability (i.e., other researchers should in principle be able to replicate the study) is a hallmark of scientific reporting.

	1	2	3	4	5	
subitem not at all important	◉	○	○	○	○	essential

Clear selection

Does your paper address subitem 5-v?

Copy and paste relevant sections from the manuscript (include quotes in quotation marks "like this" to indicate direct quotes from your manuscript), or elaborate on this item by providing additional information not in the ms, or briefly explain why the item is not applicable/relevant for your study

Not relevant as this is a secondary data analysis

5-vi) Digital preservation

Digital preservation: Provide the URL of the application, but as the intervention is likely to change or disappear over the course of the years; also make sure the intervention is archived (Internet Archive, webcitation.org, and/or publishing the source code or screenshots/videos alongside the article). As pages behind login screens cannot be archived, consider creating demo pages which are accessible without login.

	1	2	3	4	5	
subitem not at all important	◉	○	○	○	○	essential

Clear selection

Does your paper address subitem 5-vi?

Copy and paste relevant sections from the manuscript (include quotes in quotation marks "like this" to indicate direct quotes from your manuscript), or elaborate on this item by providing additional information not in the ms, or briefly explain why the item is not applicable/relevant for your study

This is more relevant to the primary study findings that have previously been published.

5-vii) Access

Access: Describe how participants accessed the application, in what setting/context, if they had to pay (or were paid) or not, whether they had to be a member of specific group. If known, describe how participants obtained “access to the platform and Internet” [1]. To ensure access for editors/reviewers/readers, consider to provide a “backdoor” login account or demo mode for reviewers/readers to explore the application (also important for archiving purposes, see vi).

	1	2	3	4	5	
subitem not at all important	○	◉	○	○	○	essential

Clear selection

Does your paper address subitem 5-vii? *

Copy and paste relevant sections from the manuscript (include quotes in quotation marks "like this" to indicate direct quotes from your manuscript), or elaborate on this item by providing additional information not in the ms, or briefly explain why the item is not applicable/relevant for your study

This was reported in Carlozzi, N.E., Troost, J.P., Sen, S., Choi, S.W., Wu, Z., Miner, J.A., Lombard, W.L., Graves, C., Sander, A.M. (In Press). Improving outcomes for care partners of individuals with traumatic brain injury: Results for a mHealth randomized control trial of the CareQOL app. Archives of Physical Medicine and Rehabilitation.

5-viii) Mode of delivery, features/functionalities/components of the intervention and comparator, and the theoretical framework

Describe mode of delivery, features/functionalities/components of the intervention and comparator, and the theoretical framework [6] used to design them (instructional strategy [1], behaviour change techniques, persuasive features, etc., see e.g., [7, 8] for terminology). This includes an in-depth description of the content (including where it is coming from and who developed it) [1],” whether [and how] it is tailored to individual circumstances and allows users to track their progress and receive feedback” [6]. This also includes a description of communication delivery channels and – if computer-mediated communication is a component – whether communication was synchronous or asynchronous [6]. It also includes information on presentation strategies [1], including page design principles, average amount of text on pages, presence of hyperlinks to other resources, etc. [1].

	1	2	3	4	5	
subitem not at all important	○	◉	○	○	○	essential

Clear selection

Does your paper address subitem 5-viii? *

Copy and paste relevant sections from the manuscript (include quotes in quotation marks "like this" to indicate direct quotes from your manuscript), or elaborate on this item by providing additional information not in the ms, or briefly explain why the item is not applicable/relevant for your study

This was reported in Carlozzi, N.E., Troost, J.P., Sen, S., Choi, S.W., Wu, Z., Miner, J.A., Lombard, W.L., Graves, C., Sander, A.M. (In Press). Improving outcomes for care partners of individuals with traumatic brain injury: Results for a mHealth randomized control trial of the CareQOL app. Archives of Physical Medicine and Rehabilitation.

In this manuscript:
Study Procedures
Participants were randomized to either a self-monitoring alone arm which included completion of the daily EMA questions, baseline, monthly, and follow-up PRO surveys, and 6 months of continuous activity and sleep monitoring with a Fitbit®, or to a self-monitoring plus self-care push notifications arm which included self-monitoring plus self-care push notifications which involved a 50/50 chance each day of receiving a self-care prompt in addition to the other assessments. All participants had access to a self-monitoring dashboard (CareQOL app) that included graphical displays of the daily EMA scores as well as daily step count and sleep duration data from the Fitbit®. Several electronic data capture and management platforms were employed, including REDCap, CareQOL, Qualtrics, Fitbit®, the University of Michigan Health Information Technology and Services server, and the Google Cloud.

5-ix) Describe use parameters

Describe use parameters (e.g., intended "doses" and optimal timing for use). Clarify what instructions or recommendations were given to the user, e.g., regarding timing, frequency, heaviness of use, if any, or was the intervention used ad libitum.

	1	2	3	4	5	
subitem not at all important	(●)	( )	( )	( )	( )	essential

Clear selection

Does your paper address subitem 5-ix?

Copy and paste relevant sections from the manuscript (include quotes in quotation marks "like this" to indicate direct quotes from your manuscript), or elaborate on this item by providing additional information not in the ms, or briefly explain why the item is not applicable/relevant for your study

Not relevant to the current analysis

5-x) Clarify the level of human involvement

Clarify the level of human involvement (care providers or health professionals, also technical assistance) in the e-intervention or as co-intervention (detail number and expertise of professionals involved, if any, as well as "type of assistance offered, the timing and frequency of the support, how it is initiated, and the medium by which the assistance is delivered". It may be necessary to distinguish between the level of human involvement required for the trial, and the level of human involvement required for a routine application outside of a RCT setting (discuss under item 21 – generalizability).

	1	2	3	4	5	
subitem not at all important	(●)	( )	( )	( )	( )	essential

Clear selection

Does your paper address subitem 5-x?

Copy and paste relevant sections from the manuscript (include quotes in quotation marks "like this" to indicate direct quotes from your manuscript), or elaborate on this item by providing additional information not in the ms, or briefly explain why the item is not applicable/relevant for your study

This was a fully remote trial; details for the protocol are published elsewhere.

Carlozzi NE, Sander AM, Choi SW, et al. Improving outcomes for care partners of persons with traumatic brain injury: Protocol for a randomized control trial of a just-in-time-adaptive self-management intervention. PLoS One. 2022;17(6):e0268726.

5-xi) Report any prompts/reminders used

Report any prompts/reminders used: Clarify if there were prompts (letters, emails, phone calls, SMS) to use the application, what triggered them, frequency etc. It may be necessary to distinguish between the level of prompts/reminders required for the trial, and the level of prompts/reminders for a routine application outside of a RCT setting (discuss under item 21 – generalizability).

	1	2	3	4	5	
subitem not at all important	○	◉	○	○	○	essential

Clear selection

Does your paper address subitem 5-xi? *

Copy and paste relevant sections from the manuscript (include quotes in quotation marks "like this" to indicate direct quotes from your manuscript), or elaborate on this item by providing additional information not in the ms, or briefly explain why the item is not applicable/relevant for your study

Previously published in: Carlozzi, N.E., Troost, J.P., Sen, S., Choi, S.W., Wu, Z., Miner, J.A., Lombard, W.L., Graves, C., Sander, A.M. (In Press). Improving outcomes for care partners of individuals with traumatic brain injury: Results for a mHealth randomized control trial of the CareQOL app. Archives of Physical Medicine and Rehabilitation.

5-xii) Describe any co-interventions (incl. training/support)

Describe any co-interventions (incl. training/support): Clearly state any interventions that are provided in addition to the targeted eHealth intervention, as ehealth intervention may not be designed as stand-alone intervention. This includes training sessions and support [1]. It may be necessary to distinguish between the level of training required for the trial, and the level of training for a routine application outside of a RCT setting (discuss under item 21 – generalizability.

	1	2	3	4	5	
subitem not at all important	◉	○	○	○	○	essential

Clear selection

Does your paper address subitem 5-xii? *

Copy and paste relevant sections from the manuscript (include quotes in quotation marks "like this" to indicate direct quotes from your manuscript), or elaborate on this item by providing additional information not in the ms, or briefly explain why the item is not applicable/relevant for your study

Not relevant to the current trial

6a) Completely defined pre-specified primary and secondary outcome measures, including how and when they were assessed

Does your paper address CONSORT subitem 6a? *

Copy and paste relevant sections from the manuscript (include quotes in quotation marks "like this" to indicate direct quotes from your manuscript), or elaborate on this item by providing additional information not in the ms, or briefly explain why the item is not applicable/relevant for your study

Published in: Carlozzi NE, Sander AM, Choi SW, et al. Improving outcomes for care partners of persons with traumatic brain injury: Protocol for a randomized control trial of a just-in-time-adaptive self-management intervention. PLoS One. 2022;17(6):e0268726.

6a-i) Online questionnaires: describe if they were validated for online use and apply CHERRIES items to describe how the questionnaires were designed/deployed

If outcomes were obtained through online questionnaires, describe if they were validated for online use and apply CHERRIES items to describe how the questionnaires were designed/deployed [9].

	1	2	3	4	5	
subitem not at all important	○	○	◉	○	○	essential

Clear selection

Does your paper address subitem 6a-i?

Copy and paste relevant sections from manuscript text

A detailed description of the study protocol is provided elsewhere.25 Briefly, participants completed a baseline assessment assessing demographic variables and measures of the care recipient's functional and emotional status (Supervision Rating Scale [SRS],26 Mayo-Portland Adaptability Inventory-Fourth Edition [MPAI-4],27 and the Posttraumatic Stress Disorder Checklist for DSM-5 [PCL-5]28) and 12 HRQOL PROs (Caregiver Strain,29, 30 Caregiver-Specific Anxiety,30, 31 PROMIS Sleep-Related Impairment,32 PROMIS Fatigue,32 Anxiety,32 Depression,32 Anger,32 Self-Efficacy-General,33 Positive Affect and Well-Being,32 Perceived Stress,33 Ability to Participate in Social Roles and Activities,32 and Global Health34). This was followed by a 6-month home monitoring period that included 3 daily EMA questions (single-item assessments of Caregiver Strain,29, 30 PROMIS Anxiety32 and PROMIS Depression32), monthly surveys (again assessing the 12 HRQOL domains) as well as continuous monitoring of physical activity and sleep monitoring using a Fitbit.® The 3- and 6-month follow-up HRQOL PROs were identical to the end of month PROs. In addition, a feasibility and acceptability survey was also administered at the end of month 6.21

Additional detail published in: Carlozzi NE, Sander AM, Choi SW, et al. Improving outcomes for care partners of persons with traumatic brain injury: Protocol for a randomized control trial of a just-in-time-adaptive self-management intervention. PLoS One. 2022;17(6):e0268726.

6a-ii) Describe whether and how "use" (including intensity of use/dosage) was defined/measured/monitored

Describe whether and how "use" (including intensity of use/dosage) was defined/measured/monitored (logins, logfile analysis, etc.). Use/adoption metrics are important process outcomes that should be reported in any ehealth trial.

	1	2	3	4	5	
subitem not at all important	○	○	●	○	○	essential

Clear selection

Does your paper address subitem 6a-ii?

Copy and paste relevant sections from manuscript text

Participants were randomized to either a self-monitoring alone arm which included completion of the daily EMA questions, baseline, monthly, and follow-up PRO surveys, and 6 months of continuous activity and sleep monitoring with a Fitbit®, or to a self-monitoring plus self-care push notifications arm which included self-monitoring plus self-care push notifications which involved a 50/50 chance each day of receiving a self-care prompt in addition to the other assessments. All participants had access to a self-monitoring dashboard (CareQOL app) that included graphical displays of the daily EMA scores as well as daily step count and sleep duration data from the Fitbit®.

6a-iii) Describe whether, how, and when qualitative feedback from participants was obtained

Describe whether, how, and when qualitative feedback from participants was obtained (e.g., through emails, feedback forms, interviews, focus groups).

	1	2	3	4	5	
subitem not at all important	(●)	( )	( )	( )	( )	essential

Clear selection

Does your paper address subitem 6a-iii?

Copy and paste relevant sections from manuscript text

We did not collect qualitative feedback in this study--that was in complementary study whose findings are currently under review at a different journal.

6b) Any changes to trial outcomes after the trial commenced, with reasons

Does your paper address CONSORT subitem 6b? *

Copy and paste relevant sections from the manuscript (include quotes in quotation marks "like this" to indicate direct quotes from your manuscript), or elaborate on this item by providing additional information not in the ms, or briefly explain why the item is not applicable/relevant for your study

There were not any changes in outcomes after the trial commenced.

7a) How sample size was determined

NPT: When applicable, details of whether and how the clustering by care provides or centers was addressed

7a-i) Describe whether and how expected attrition was taken into account when calculating the sample size

Describe whether and how expected attrition was taken into account when calculating the sample size.

	1	2	3	4	5	
subitem not at all important	(●)	○	○	○	○	essential

Clear selection

Does your paper address subitem 7a-i?

Copy and paste relevant sections from manuscript title (include quotes in quotation marks "like this" to indicate direct quotes from your manuscript), or elaborate on this item by providing additional information not in the ms, or briefly explain why the item is not applicable/relevant for your study

Published in: Carlozzi NE, Sander AM, Choi SW, et al. Improving outcomes for care partners of persons with traumatic brain injury: Protocol for a randomized control trial of a just-in-time-adaptive self-management intervention. PLoS One. 2022;17(6):e0268726.

7b) When applicable, explanation of any interim analyses and stopping guidelines

Does your paper address CONSORT subitem 7b? *

Copy and paste relevant sections from the manuscript (include quotes in quotation marks "like this" to indicate direct quotes from your manuscript), or elaborate on this item by providing additional information not in the ms, or briefly explain why the item is not applicable/relevant for your study

This was not relevant to this analysis.

8a) Method used to generate the random allocation sequence

NPT: When applicable, how care providers were allocated to each trial group

Does your paper address CONSORT subitem 8a? *

Copy and paste relevant sections from the manuscript (include quotes in quotation marks "like this" to indicate direct quotes from your manuscript), or elaborate on this item by providing additional information not in the ms, or briefly explain why the item is not applicable/relevant for your study

Participants were randomized to either a self-monitoring alone arm which included completion of the daily EMA questions, baseline, monthly, and follow-up PRO surveys, and 6 months of continuous activity and sleep monitoring with a Fitbit®, or to a self-monitoring plus self-care push notifications arm which included self-monitoring plus self-care push notifications which involved a 50/50 chance each day of receiving a self-care prompt in addition to the other assessments.

8b) Type of randomisation; details of any restriction (such as blocking and block size)

Does your paper address CONSORT subitem 8b? *

Copy and paste relevant sections from the manuscript (include quotes in quotation marks "like this" to indicate direct quotes from your manuscript), or elaborate on this item by providing additional information not in the ms, or briefly explain why the item is not applicable/relevant for your study

participants were block-randomized at a 1:1 rate to one of the two study arms

9) Mechanism used to implement the random allocation sequence (such as sequentially numbered containers), describing any steps taken to conceal the sequence until interventions were assigned

Does your paper address CONSORT subitem 9? *

Copy and paste relevant sections from the manuscript (include quotes in quotation marks "like this" to indicate direct quotes from your manuscript), or elaborate on this item by providing additional information not in the ms, or briefly explain why the item is not applicable/relevant for your study

We did not employ this type of randomization

10) Who generated the random allocation sequence, who enrolled participants, and who assigned participants to interventions

Does your paper address CONSORT subitem 10? *

Copy and paste relevant sections from the manuscript (include quotes in quotation marks "like this" to indicate direct quotes from your manuscript), or elaborate on this item by providing additional information not in the ms, or briefly explain why the item is not applicable/relevant for your study

We did not employ this type of randomization

11a) If done, who was blinded after assignment to interventions (for example, participants, care providers, those assessing outcomes) and how
NPT: Whether or not administering co-interventions were blinded to group assignment

11a-i) Specify who was blinded, and who wasn't

Specify who was blinded, and who wasn't. Usually, in web-based trials it is not possible to blind the participants [1, 3] (this should be clearly acknowledged), but it may be possible to blind outcome assessors, those doing data analysis or those administering co-interventions (if any).

	1	2	3	4	5	
subitem not at all important	(•)	( )	( )	( )	( )	essential

Clear selection

Does your paper address subitem 11a-i? *

Copy and paste relevant sections from the manuscript (include quotes in quotation marks "like this" to indicate direct quotes from your manuscript), or elaborate on this item by providing additional information not in the ms, or briefly explain why the item is not applicable/relevant for your study

We did not employ blinding in this study

11a-ii) Discuss e.g., whether participants knew which intervention was the "intervention of interest" and which one was the "comparator"

Informed consent procedures (4a-ii) can create biases and certain expectations - discuss e.g., whether participants knew which intervention was the "intervention of interest" and which one was the "comparator".

	1	2	3	4	5	
subitem not at all important	○	○	◉	○	○	essential

Clear selection

Does your paper address subitem 11a-ii?

Copy and paste relevant sections from the manuscript (include quotes in quotation marks "like this" to indicate direct quotes from your manuscript), or elaborate on this item by providing additional information not in the ms, or briefly explain why the item is not applicable/relevant for your study

During the informed consent process, participants were told about the different study arms.

11b) If relevant, description of the similarity of interventions

(this item is usually not relevant for ehealth trials as it refers to similarity of a placebo or sham intervention to a active medication/intervention)

Does your paper address CONSORT subitem 11b? *

Copy and paste relevant sections from the manuscript (include quotes in quotation marks "like this" to indicate direct quotes from your manuscript), or elaborate on this item by providing additional information not in the ms, or briefly explain why the item is not applicable/relevant for your study

This is not relevant to the current study

12a) Statistical methods used to compare groups for primary and secondary outcomes
NPT: When applicable, details of whether and how the clustering by care providers or centers was addressed

Does your paper address CONSORT subitem 12a? *

Copy and paste relevant sections from the manuscript (include quotes in quotation marks "like this" to indicate direct quotes from your manuscript), or elaborate on this item by providing additional information not in the ms, or briefly explain why the item is not applicable/relevant for your study

First, we examined completion rates for daily EMA questions and monthly surveys, separately by study arm (calculated as the percentage of days with data over the number of days in the study for daily, monthly, and follow-up surveys), and then conducted a series of independent sample t tests to determine whether there were differences by study arm. Next, we examined the interrelationships among different types of completion (EMA questions, monthly survey responses) and compliance (Fitbit®-based estimates of daily step count and sleep duration). We then conducted a series of linear regression analyses to determine which variables (including demographic variables; baseline PROs, SRS, MPAI-4, and PCL-5; and feasibility and acceptability questions [assessed at 6 months]) predicted completion/compliance rates.

In addition, k-means clustering was used to identify latent (i.e., “unobserved”) categorical subgroups of respondents based on their daily completion and compliance rates. The optimal number of clusters was determined based on assessment of model fit and parsimony (pseudo F statistic, approximate R-squared, cubic clustering criterion). Once class number was determined, respondents were classified into latent classes based on maximum posterior probability. We also examined whether or not different descriptive variables (care partner age, care partner gender, care recipient age, care recipient gender, care partner race, care partner ethnicity, duration providing care, time spent caregiving, relationship to care recipient, work status, SRS score, PCL score, and functional ability of the care recipient) were able to predict the identified clusters.

12a-i) Imputation techniques to deal with attrition / missing values

Imputation techniques to deal with attrition / missing values: Not all participants will use the intervention/comparator as intended and attrition is typically high in ehealth trials. Specify how participants who did not use the application or dropped out from the trial were treated in the statistical analysis (a complete case analysis is strongly discouraged, and simple imputation techniques such as LOCF may also be problematic [4]).

	1	2	3	4	5	
subitem not at all important	◉	○	○	○	○	essential

Clear selection

Does your paper address subitem 12a-i? *

Copy and paste relevant sections from the manuscript (include quotes in quotation marks "like this" to indicate direct quotes from your manuscript), or elaborate on this item by providing additional information not in the ms, or briefly explain why the item is not applicable/relevant for your study

imputation was not used; given that we were exploring completion and compliance in this paper, we provide details throughout about the completion and compliance rates

12b) Methods for additional analyses, such as subgroup analyses and adjusted analyses

Does your paper address CONSORT subitem 12b? *

Copy and paste relevant sections from the manuscript (include quotes in quotation marks "like this" to indicate direct quotes from your manuscript), or elaborate on this item by providing additional information not in the ms, or briefly explain why the item is not applicable/relevant for your study

First, we examined completion rates for daily EMA questions and monthly surveys, separately by study arm (calculated as the percentage of days with data over the number of days in the study for daily, monthly, and follow-up surveys), and then conducted a series of independent sample t tests to determine whether there were differences by study arm. Next, we examined the interrelationships among different types of completion (EMA questions, monthly survey responses) and compliance (Fitbit®-based estimates of daily step count and sleep duration). We then conducted a series of linear regression analyses to determine which variables (including demographic variables; baseline PROs, SRS, MPAI-4, and PCL-5; and feasibility and acceptability questions [assessed at 6 months]) predicted completion/compliance rates.

In addition, k-means clustering was used to identify latent (i.e., "unobserved") categorical subgroups of respondents based on their daily completion and compliance rates. The optimal number of clusters was determined based on assessment of model fit and parsimony (pseudo F statistic, approximate R-squared, cubic clustering criterion). Once class number was determined, respondents were classified into latent classes based on maximum posterior probability. We also examined whether or not different descriptive variables (care partner age, care partner gender, care recipient age, care recipient gender, care partner race, care partner ethnicity, duration providing care, time spent caregiving, relationship to care recipient, work status, SRS score, PCL score, and functional ability of the care recipient) were able to predict the identified clusters.

X26) REB/IRB Approval and Ethical Considerations [recommended as subheading under "Methods"] (not a CONSORT item)

X26-i) Comment on ethics committee approval

	1	2	3	4	5	
subitem not at all important	○	◉	○	○	○	essential

Clear selection

Does your paper address subitem X26-i?

Copy and paste relevant sections from the manuscript (include quotes in quotation marks "like this" to indicate direct quotes from your manuscript), or elaborate on this item by providing additional information not in the ms, or briefly explain why the item is not applicable/relevant for your study

This is reported in a previously published paper: This study was performed in accordance with institutional review board (IRB) approvals (IRBMED Multi-site Application Approval HUM00181282; IRBMED University of Michigan Site Application Approval HUM00186921; IRBMED Baylor College of Medicine Site Application Approval SITE0000087; Baylor College of Medicine/Memorial Hermann IRB number H-48478) and is registered with ClinicalTrials.gov (NCT04570930).

Carlozzi, N.E., Troost, J.P., Sen, S., Choi, S.W., Wu, Z., Miner, J.A., Lombard, W.L., Graves, C., Sander, A.M. (In Press). Improving outcomes for care partners of individuals with traumatic brain injury: Results for a mHealth randomized control trial of the CareQOL app. Archives of Physical Medicine and Rehabilitation.

x26-ii) Outline informed consent procedures

Outline informed consent procedures e.g., if consent was obtained offline or online (how? Checkbox, etc.?), and what information was provided (see 4a-ii). See [6] for some items to be included in informed consent documents.

	1	2	3	4	5	
subitem not at all important	○	●	○	○	○	essential

Clear selection

Does your paper address subitem X26-ii?

Copy and paste relevant sections from the manuscript (include quotes in quotation marks "like this" to indicate direct quotes from your manuscript), or elaborate on this item by providing additional information not in the ms, or briefly explain why the item is not applicable/relevant for your study

published in protocol manuscript: provide informed consent prior to completing any study assessments. Carlozzi NE, Sander AM, Choi SW, et al. Improving outcomes for care partners of persons with traumatic brain injury: Protocol for a randomized control trial of a just-in-time-adaptive self-management intervention. PLoS One. 2022;17(6):e0268726.

X26-iii) Safety and security procedures

Safety and security procedures, incl. privacy considerations, and any steps taken to reduce the likelihood or detection of harm (e.g., education and training, availability of a hotline)

	1	2	3	4	5	
subitem not at all important	○	◉	○	○	○	essential

Clear selection

Does your paper address subitem X26-iii?

Copy and paste relevant sections from the manuscript (include quotes in quotation marks "like this" to indicate direct quotes from your manuscript), or elaborate on this item by providing additional information not in the ms, or briefly explain why the item is not applicable/relevant for your study

published in protocol manuscript: Carlozzi NE, Sander AM, Choi SW, et al. Improving outcomes for care partners of persons with traumatic brain injury: Protocol for a randomized control trial of a just-in-time-adaptive self-management intervention. PLoS One. 2022;17(6):e0268726.

RESULTS

13a) For each group, the numbers of participants who were randomly assigned, received intended treatment, and were analysed for the primary outcome
NPT: The number of care providers or centers performing the intervention in each group and the number of patients treated by each care provider in each center

Does your paper address CONSORT subitem 13a? *

Copy and paste relevant sections from the manuscript (include quotes in quotation marks "like this" to indicate direct quotes from your manuscript), or elaborate on this item by providing additional information not in the ms, or briefly explain why the item is not applicable/relevant for your study

A total of 254 care partners of people with TBI participated in this study. Details describing the demographic data for the different study arms have been published previously (see Carlozzi et al.23). Table 2 provides an abbreviated summary of the demographic data for the full sample.

13b) For each group, losses and exclusions after randomisation, together with reasons

Does your paper address CONSORT subitem 13b? (NOTE: Preferably, this is shown in a CONSORT flow diagram) *

Copy and paste relevant sections from the manuscript (include quotes in quotation marks "like this" to indicate direct quotes from your manuscript), or elaborate on this item by providing additional information not in the ms, or briefly explain why the item is not applicable/relevant for your study

A total of 254 care partners of people with TBI participated in this study. Details describing the demographic data for the different study arms have been published previously (see Carlozzi et al.; Carlozzi, N.E., Troost, J.P., Sen, S., Choi, S.W., Wu, Z., Miner, J.A., Lombard, W.L., Graves, C., Sander, A.M. (In Press). Improving outcomes for care partners of individuals with traumatic brain injury: Results for a mHealth randomized control trial of the CareQOL app. Archives of Physical Medicine and Rehabilitation). Table 2 provides an abbreviated summary of the demographic data for the full sample.

13b-i) Attrition diagram

Strongly recommended: An attrition diagram (e.g., proportion of participants still logging in or using the intervention/comparator in each group plotted over time, similar to a survival curve) or other figures or tables demonstrating usage/dose/engagement.

	1	2	3	4	5	
subitem not at all important	◉	○	○	○	○	essential

Clear selection

Does your paper address subitem 13b-i?

Copy and paste relevant sections from the manuscript or cite the figure number if applicable (include quotes in quotation marks "like this" to indicate direct quotes from your manuscript), or elaborate on this item by providing additional information not in the ms, or briefly explain why the item is not applicable/relevant for your study

This was reported in a previous publication: Carlozzi, N.E., Troost, J.P., Sen, S., Choi, S.W., Wu, Z., Miner, J.A., Lombard, W.L., Graves, C., Sander, A.M. (In Press). Improving outcomes for care partners of individuals with traumatic brain injury: Results for a mHealth randomized control trial of the CareQOL app. Archives of Physical Medicine and Rehabilitation.

14a) Dates defining the periods of recruitment and follow-up

Does your paper address CONSORT subitem 14a? *

Copy and paste relevant sections from the manuscript (include quotes in quotation marks "like this" to indicate direct quotes from your manuscript), or elaborate on this item by providing additional information not in the ms, or briefly explain why the item is not applicable/relevant for your study

previously reported in: Carlozzi, N.E., Troost, J.P., Sen, S., Choi, S.W., Wu, Z., Miner, J.A., Lombard, W.L., Graves, C., Sander, A.M. (In Press). Improving outcomes for care partners of individuals with traumatic brain injury: Results for a mHealth randomized control trial of the CareQOL app. Archives of Physical Medicine and Rehabilitation.

14a-i) Indicate if critical “secular events” fell into the study period

Indicate if critical “secular events” fell into the study period, e.g., significant changes in Internet resources available or “changes in computer hardware or Internet delivery resources”

	1	2	3	4	5	
subitem not at all important	(●)	○	○	○	○	essential

Clear selection

Does your paper address subitem 14a-i?

Copy and paste relevant sections from the manuscript (include quotes in quotation marks "like this" to indicate direct quotes from your manuscript), or elaborate on this item by providing additional information not in the ms, or briefly explain why the item is not applicable/relevant for your study

Not relevant as the study was fully remote

14b) Why the trial ended or was stopped (early)

Does your paper address CONSORT subitem 14b? *

Copy and paste relevant sections from the manuscript (include quotes in quotation marks "like this" to indicate direct quotes from your manuscript), or elaborate on this item by providing additional information not in the ms, or briefly explain why the item is not applicable/relevant for your study

The trial ended when we hit our proposed completion targets: see clinicaltrials.gov posting.

15) A table showing baseline demographic and clinical characteristics for each group

NPT: When applicable, a description of care providers (case volume, qualification, expertise, etc.) and centers (volume) in each group

Does your paper address CONSORT subitem 15? *

Copy and paste relevant sections from the manuscript (include quotes in quotation marks "like this" to indicate direct quotes from your manuscript), or elaborate on this item by providing additional information not in the ms, or briefly explain why the item is not applicable/relevant for your study

Reported in Table 2:

Sample descriptive data

	M (SD) or N (%)
Age, mean (SD)	52.0 (14.7)
Age of person cared for, mean (SD)	43.0 (17.7)
Sex (female), n (%)	201 (79)
Sex of person cared for (female), n (%)	68 (27)
Race	
American Indian or Alaska Native	3 (1)
Asian	10 (4)
Native Hawaiian or Other Pacific Islander	1 (1)
Black or African American	36 (14)
White or Caucasian	195 (77)
More than one race	8 (3)
Missing	1 (1)
Hispanic Ethnicity, n (%)	
Length of caregiving, mean (SD)	6.5 (5.7)
Relation to caregiver	
Partner	104 (41)
Child	38 (15)
Parent	81 (32)
Sibling	20 (8)
Other family	3 (1)
Friend	3 (1)
Missing	5 (2)
Same household	
Yes, all the time	182 (72)
Yes, but only a few days a week	13 (5)
No	59 (23)
Work status	
Employed full time (at least 40 hours/week)	111 (44)
Employed part-time	29 (11)
Homemaker	17 (7)
Student	11 (4)
Retired	56 (22)
Retired early due to disability	2 (1)
Unemployed <1 year, and LOOKING for work	4 (2)
Unemployed <1 year, NOT LOOKING for work	1 (1)
Unemployed >1 year, LOOKING for work	3 (1)
Unemployed >1 year, NOT LOOKING for work	7 (3)
Unable to work/disabled	8 (3)
Other	5 (2)
How much assistance does the person you care for require from you to complete activities of daily living due to problems resulting from his/her TBI? (0-10)	6.3 (2.4)
Supervision Rating Scale (SRS), mean (SD) range	4.0 (3.1) 1-12
Posttraumatic stress symptoms (PCL-5) mean (SD) range	20.0 (15.6) 0-74
[illegible]	

Time spent caregiving

1 to 2 hours per day or less 121 (48)

3 to 4 hours per day (i.e., half of a working day) 54 (21)

5 to 8 hours per day (i.e., full working day) 23 (9)

9 to 12 hours per day 13 (5)

>12 hours per day or round-the-clock care 43 (17)

15-i) Report demographics associated with digital divide issues

In ehealth trials it is particularly important to report demographics associated with digital divide issues, such as age, education, gender, social-economic status, computer/Internet/ehealth literacy of the participants, if known.

	1	2	3	4	5	
subitem not at all important	○	○	◉	○	○	essential

Clear selection

Does your paper address subitem 15-i? *

Copy and paste relevant sections from the manuscript (include quotes in quotation marks "like this" to indicate direct quotes from your manuscript), or elaborate on this item by providing additional information not in the ms, or briefly explain why the item is not applicable/relevant for your study

Reported in Table 2: Sample descriptive data

	M (SD) or N (%)
Age, mean (SD)	52.0 (14.7)
Age of person cared for, mean (SD)	43.0 (17.7)
Sex (female), n (%)	201 (79)
Sex of person cared for (female), n (%)	68 (27)
Race	
American Indian or Alaska Native	3 (1)
Asian	10 (4)
Native Hawaiian or Other Pacific Islander	1 (1)
Black or African American	36 (14)
White or Caucasian	195 (77)
More than one race	8 (3)
Missing	1 (1)
Hispanic Ethnicity, n (%)	
Length of caregiving, mean (SD)	6.5 (5.7)
Relation to caregiver	
Partner	104 (41)
Child	38 (15)
Parent	81 (32)
Sibling	20 (8)
Other family	3 (1)
Friend	3 (1)
Missing	5 (2)
Same household	
Yes, all the time	182 (72)
Yes, but only a few days a week	13 (5)
No	59 (23)
Work status	
Employed full time (at least 40 hours/week)	111 (44)
Employed part-time	29 (11)
Homemaker	17 (7)
Student	11 (4)
Retired	56 (22)
Retired early due to disability	2 (1)
Unemployed <1 year, and LOOKING for work	4 (2)
Unemployed <1 year, NOT LOOKING for work	1 (1)
Unemployed >1 year, LOOKING for work	3 (1)
Unemployed >1 year, NOT LOOKING for work	7 (3)
Unable to work/disabled	8 (3)
Other	5 (2)
How much assistance does the person you care for require from you to complete activities of daily living due to problems resulting from his/her TBI? (0-10)	6.3 (2.4)
Supervision Rating Scale (SRS), mean (SD) range	4.0 (3.1) 1-12
Posttraumatic stress symptoms (PCL-5) mean (SD) range	20.0 (15.6) 0-74
Time spent caregiving	
1 to 2 hours per day or less	121 (48)
[illegible]	[illegible]

3 to 4 hours per day (i.e., half of a working day) 54 (21)
5 to 8 hours per day (i.e., full working day) 23 (9)
9 to 12 hours per day 13 (5)
>12 hours per day or round-the-clock care 43 (17)

16) For each group, number of participants (denominator) included in each analysis and whether the analysis was by original assigned groups

16-i) Report multiple “denominators” and provide definitions

Report multiple “denominators” and provide definitions: Report N’s (and effect sizes) “across a range of study participation [and use] thresholds” [1], e.g., N exposed, N consented, N used more than x times, N used more than y weeks, N participants “used” the intervention/comparator at specific pre-defined time points of interest (in absolute and relative numbers per group). Always clearly define “use” of the intervention.

	1	2	3	4	5	
subitem not at all important	○	○	◉	○	○	essential

Clear selection

Does your paper address subitem 16-i? *

Copy and paste relevant sections from the manuscript (include quotes in quotation marks "like this" to indicate direct quotes from your manuscript), or elaborate on this item by providing additional information not in the ms, or briefly explain why the item is not applicable/relevant for your study

A total of 254 care partners of people with TBI participated in this study. Details describing the demographic data for the different study arms have been published previously (see Carlozzi et al.23). Table 2 provides an abbreviated summary of the demographic data for the full sample.

Overall compliance for the different aspects of the study was high (see Tables 3 & 4). Specifically, on average, the full-sample daily EMA completion rate was 84%, Fitbit®-based step count compliance was 90%, and Fitbit®-based sleep duration compliance was 75%; there was no difference between the study arms for daily completion and compliance rates (Table 3). Completion rates for monthly and follow-up surveys were even higher, with average end-of-month completion rates ranging from 94% to 98%, and follow-up completion rates of 92% for both 3-month and 6-month post time points; again, these rates did not differ by study arm (Table 4). Compliance rates were moderately correlated for the two Fitbit®-based measures ($r = 0.65$), and the magnitude of the correlations were less robust between Fitbit®-based compliance data and the daily EMAs ($r = 0.38$ between steps and EMAs and $r = 0.29$ between sleep and EMAs).

Linear regression analyses indicated that: 1) several feasibility and acceptability questions related to ease, satisfaction, and burden of completing the EMAs predicted daily EMA completion rates; 2) race, relation to caregiver, and several of the feasibility and acceptability questions related to using the Fitbit® predicted daily Fitbit®-based step count compliance rates; and 3) race, relationship status, eligibility rating and some of the feasibility and acceptability questions related to using the Fitbit® predicted daily Fitbit®-based sleep duration compliance rates (see Appendix 1). More specifically, positive perceptions about feasibility and acceptability were related to better completion and compliance rates for EMAs and monthly surveys, steps, and sleep; being Black was associated with lower Fitbit®-based compliance rates for both steps and sleep; being a friend or other family member was associated with lower Fitbit®-based compliance rates for daily steps; being single and caring for someone with more functional deficits was associated with lower Fitbit®-based compliance rates for sleep (Table 5).

Results from the cluster analysis indicated that the data was best represented by three groups (see Figure 1 and Appendix 2): 1) the “high-compliance group-all data” cluster, where compliance across all data types (EMA and survey PROs, steps, and sleep) was high (72% of participants); 2) the “high-compliance group-PROs and steps only” cluster, where compliance was high for EMA and survey PROs and Fitbit®-based compliance rates for steps, but not for sleep (22% of participants); and 3) the “moderate PRO compliance, low Fitbit® compliance group” where monthly survey completion was good, but not excellent, and Fitbit® compliance for both steps and sleep was low (7% of people). Cluster membership was predicted by both race ($p<.001$), with a larger proportion of white caregivers relative to the other racial groups in cluster 1 (high compliance group-all data) relative to the other two clusters (high compliance group-PROs and steps only” and moderate PRO and low Fitbit® compliance group), and relationship to the care recipient ($p = .001$), with the moderate PRO and low Fitbit® compliance group having a higher proportion of parents and a lower proportion of partners than the other two clusters, but not to other demographic factors or other characteristics (Table 6).

[illegible]

Also see Tables 2-6

16-ii) Primary analysis should be intent-to-treat

Primary analysis should be intent-to-treat, secondary analyses could include comparing only "users", with the appropriate caveats that this is no longer a randomized sample (see 18-i).

	1	2	3	4	5	
subitem not at all important	◉	○	○	○	○	essential

Clear selection

Does your paper address subitem 16-ii?

Copy and paste relevant sections from the manuscript (include quotes in quotation marks "like this" to indicate direct quotes from your manuscript), or elaborate on this item by providing additional information not in the ms, or briefly explain why the item is not applicable/relevant for your study

This is a secondary analysis --this information was previously published in: Carlozzi, N.E., Troost, J.P., Sen, S., Choi, S.W., Wu, Z., Miner, J.A., Lombard, W.L., Graves, C., Sander, A.M. (In Press). Improving outcomes for care partners of individuals with traumatic brain injury: Results for a mHealth randomized control trial of the CareQOL app. Archives of Physical Medicine and Rehabilitation.

17a) For each primary and secondary outcome, results for each group, and the estimated effect size and its precision (such as 95% confidence interval)

Does your paper address CONSORT subitem 17a? *

Copy and paste relevant sections from the manuscript (include quotes in quotation marks "like this" to indicate direct quotes from your manuscript), or elaborate on this item by providing additional information not in the ms, or briefly explain why the item is not applicable/relevant for your study

This is not relevant to the analyses in the current paper

17a-i) Presentation of process outcomes such as metrics of use and intensity of use

In addition to primary/secondary (clinical) outcomes, the presentation of process outcomes such as metrics of use and intensity of use (dose, exposure) and their operational definitions is critical. This does not only refer to metrics of attrition (13-b) (often a binary variable), but also to more continuous exposure metrics such as "average session length". These must be accompanied by a technical description how a metric like a "session" is defined (e.g., timeout after idle time) [1] (report under item 6a).

	1	2	3	4	5	
subitem not at all important	○	◉	○	○	○	essential

Clear selection

Does your paper address subitem 17a-i?

Copy and paste relevant sections from the manuscript (include quotes in quotation marks "like this" to indicate direct quotes from your manuscript), or elaborate on this item by providing additional information not in the ms, or briefly explain why the item is not applicable/relevant for your study

Overall compliance for the different aspects of the study was high (see Tables 3 & 4). Specifically, on average, the full-sample daily EMA completion rate was 84%, Fitbit®-based step count compliance was 90%, and Fitbit®-based sleep duration compliance was 75%; there was no difference between the study arms for daily completion and compliance rates (Table 3). Completion rates for monthly and follow-up surveys were even higher, with average end-of-month completion rates ranging from 94% to 98%, and follow-up completion rates of 92% for both 3-month and 6-month post time points; again, these rates did not differ by study arm (Table 4). Compliance rates were moderately correlated for the two Fitbit®-based measures (r = 0.65), and the magnitude of the correlations were less robust between Fitbit®-based compliance data and the daily EMAs (r = 0.38 between steps and EMAs and r = 0.29 between sleep and EMAs).

Linear regression analyses indicated that: 1) several feasibility and acceptability questions related to ease, satisfaction, and burden of completing the EMAs predicted daily EMA completion rates; 2) race, relation to caregiver, and several of the feasibility and acceptability questions related to using the Fitbit® predicted daily Fitbit®-based step count compliance rates; and 3) race, relationship status, eligibility rating and some of the feasibility and acceptability questions related to using the Fitbit® predicted daily Fitbit®-based sleep duration compliance rates (see Appendix 1). More specifically, positive perceptions about feasibility and acceptability were related to better completion and compliance rates for EMAs and monthly surveys, steps, and sleep; being Black was associated with lower Fitbit®-based compliance rates for both steps and sleep; being a friend or other family member was associated with lower Fitbit®-based compliance rates for daily steps; being single and caring for someone with more functional deficits was associated with lower Fitbit®-based compliance rates for sleep (Table 5).

17b) For binary outcomes, presentation of both absolute and relative effect sizes is recommended

Does your paper address CONSORT subitem 17b? *

Copy and paste relevant sections from the manuscript (include quotes in quotation marks "like this" to indicate direct quotes from your manuscript), or elaborate on this item by providing additional information not in the ms, or briefly explain why the item is not applicable/relevant for your study

This is not relevant to the analyses that are reported

18) Results of any other analyses performed, including subgroup analyses and adjusted analyses, distinguishing pre-specified from exploratory

Does your paper address CONSORT subitem 18? *

Copy and paste relevant sections from the manuscript (include quotes in quotation marks "like this" to indicate direct quotes from your manuscript), or elaborate on this item by providing additional information not in the ms, or briefly explain why the item is not applicable/relevant for your study

Overall compliance for the different aspects of the study was high (see Tables 3 & 4). Specifically, on average, the full-sample daily EMA completion rate was 84%, Fitbit®-based step count compliance was 90%, and Fitbit®-based sleep duration compliance was 75%; there was no difference between the study arms for daily completion and compliance rates (Table 3). Completion rates for monthly and follow-up surveys were even higher, with average end-of-month completion rates ranging from 94% to 98%, and follow-up completion rates of 92% for both 3-month and 6-month post time points; again, these rates did not differ by study arm (Table 4). Compliance rates were moderately correlated for the two Fitbit®-based measures ($r = 0.65$), and the magnitude of the correlations were less robust between Fitbit®-based compliance data and the daily EMAs ($r = 0.38$ between steps and EMAs and $r = 0.29$ between sleep and EMAs).

Linear regression analyses indicated that: 1) several feasibility and acceptability questions related to ease, satisfaction, and burden of completing the EMAs predicted daily EMA completion rates; 2) race, relation to caregiver, and several of the feasibility and acceptability questions related to using the Fitbit® predicted daily Fitbit®-based step count compliance rates; and 3) race, relationship status, eligibility rating and some of the feasibility and acceptability questions related to using the Fitbit® predicted daily Fitbit®-based sleep duration compliance rates (see Appendix 1). More specifically, positive perceptions about feasibility and acceptability were related to better completion and compliance rates for EMAs and monthly surveys, steps, and sleep; being Black was associated with lower Fitbit®-based compliance rates for both steps and sleep; being a friend or other family member was associated with lower Fitbit®-based compliance rates for daily steps; being single and caring for someone with more functional deficits was associated with lower Fitbit®-based compliance rates for sleep (Table 5).

Results from the cluster analysis indicated that the data was best represented by three groups (see Figure 1 and Appendix 2): 1) the “high-compliance group-all data” cluster, where compliance across all data types (EMA and survey PROs, steps, and sleep) was high (72% of participants); 2) the “high-compliance group-PROs and steps only” cluster, where compliance was high for EMA and survey PROs and Fitbit®-based compliance rates for steps, but not for sleep (22% of participants); and 3) the “moderate PRO compliance, low Fitbit® compliance group” where monthly survey completion was good, but not excellent, and Fitbit® compliance for both steps and sleep was low (7% of people). Cluster membership was predicted by both race ($p<.001$), with a larger proportion of white caregivers relative to the other racial groups in cluster 1 (high compliance group-all data) relative to the other two clusters (high compliance group-PROs and steps only” and moderate PRO and low Fitbit® compliance group), and relationship to the care recipient ($p = .001$), with the moderate PRO and low Fitbit® compliance group having a higher proportion of parents and a lower proportion of partners than the other two clusters, but not to other demographic factors or other characteristics (Table 6).

18-i) Subgroup analysis of comparing only users

A subgroup analysis of comparing only users is not uncommon in ehealth trials, but if done, it must be stressed that this is a self-selected sample and no longer an unbiased sample from a randomized trial (see 16-iii).

	1	2	3	4	5	
subitem not at all important	○	○	◉	○	○	essential

Clear selection

Does your paper address subitem 18-i?

Copy and paste relevant sections from the manuscript (include quotes in quotation marks "like this" to indicate direct quotes from your manuscript), or elaborate on this item by providing additional information not in the ms, or briefly explain why the item is not applicable/relevant for your study

Linear regression analyses indicated that: 1) several feasibility and acceptability questions related to ease, satisfaction, and burden of completing the EMAs predicted daily EMA completion rates; 2) race, relation to caregiver, and several of the feasibility and acceptability questions related to using the Fitbit® predicted daily Fitbit®-based step count compliance rates; and 3) race, relationship status, eligibility rating and some of the feasibility and acceptability questions related to using the Fitbit® predicted daily Fitbit®-based sleep duration compliance rates (see Appendix 1). More specifically, positive perceptions about feasibility and acceptability were related to better completion and compliance rates for EMAs and monthly surveys, steps, and sleep; being Black was associated with lower Fitbit®-based compliance rates for both steps and sleep; being a friend or other family member was associated with lower Fitbit®-based compliance rates for daily steps; being single and caring for someone with more functional deficits was associated with lower Fitbit®-based compliance rates for sleep (Table 5).

Results from the cluster analysis indicated that the data was best represented by three groups (see Figure 1 and Appendix 2): 1) the "high-compliance group-all data" cluster, where compliance across all data types (EMA and survey PROs, steps, and sleep) was high (72% of participants); 2) the "high-compliance group-PROs and steps only" cluster, where compliance was high for EMA and survey PROs and Fitbit®-based compliance rates for steps, but not for sleep (22% of participants); and 3) the "moderate PRO compliance, low Fitbit® compliance group" where monthly survey completion was good, but not excellent, and Fitbit® compliance for both steps and sleep was low (7% of people). Cluster membership was predicted by both race ($p<.001$), with a larger proportion of white caregivers relative to the other racial groups in cluster 1 (high compliance group-all data) relative to the other two clusters (high compliance group-PROs and steps only" and moderate PRO and low Fitbit® compliance group), and relationship to the care recipient ($p = .001$), with the moderate PRO and low Fitbit® compliance group having a higher proportion of parents and a lower proportion of partners than the other two clusters, but not to other demographic factors or other characteristics (Table 6).

19) All important harms or unintended effects in each group

(for specific guidance see CONSORT for harms)

Does your paper address CONSORT subitem 19? *

Copy and paste relevant sections from the manuscript (include quotes in quotation marks "like this" to indicate direct quotes from your manuscript), or elaborate on this item by providing additional information not in the ms, or briefly explain why the item is not applicable/relevant for your study

Previously published in: Carlozzi, N.E., Troost, J.P., Sen, S., Choi, S.W., Wu, Z., Miner, J.A., Lombard, W.L., Graves, C., Sander, A.M. (In Press). Improving outcomes for care partners of individuals with traumatic brain injury: Results for a mHealth randomized control trial of the CareQOL app. Archives of Physical Medicine and Rehabilitation.

19-i) Include privacy breaches, technical problems

Include privacy breaches, technical problems. This does not only include physical “harm” to participants, but also incidents such as perceived or real privacy breaches [1], technical problems, and other unexpected/unintended incidents. “Unintended effects” also includes unintended positive effects [2].

	1	2	3	4	5	
subitem not at all important	(●)	( )	( )	( )	( )	essential

Clear selection

Does your paper address subitem 19-i?

Copy and paste relevant sections from the manuscript (include quotes in quotation marks "like this" to indicate direct quotes from your manuscript), or elaborate on this item by providing additional information not in the ms, or briefly explain why the item is not applicable/relevant for your study

Previously published: Carlozzi, N.E., Troost, J.P., Sen, S., Choi, S.W., Wu, Z., Miner, J.A., Lombard, W.L., Graves, C., Sander, A.M. (In Press). Improving outcomes for care partners of individuals with traumatic brain injury: Results for a mHealth randomized control trial of the CareQOL app. Archives of Physical Medicine and Rehabilitation.

19-ii) Include qualitative feedback from participants or observations from staff/researchers

Include qualitative feedback from participants or observations from staff/researchers, if available, on strengths and shortcomings of the application, especially if they point to unintended/unexpected effects or uses. This includes (if available) reasons for why people did or did not use the application as intended by the developers.

	1	2	3	4	5	
subitem not at all important	(selected)	○	○	○	○	essential

Clear selection

Does your paper address subitem 19-ii?

Copy and paste relevant sections from the manuscript (include quotes in quotation marks "like this" to indicate direct quotes from your manuscript), or elaborate on this item by providing additional information not in the ms, or briefly explain why the item is not applicable/relevant for your study

This data was collected as a part of a complementary study and findings are currently under peer review with a different journal

DISCUSSION

22) Interpretation consistent with results, balancing benefits and harms, and considering other relevant evidence

NPT: In addition, take into account the choice of the comparator, lack of or partial blinding, and unequal expertise of care providers or centers in each group

22-i) Restate study questions and summarize the answers suggested by the data, starting with primary outcomes and process outcomes (use)

Restate study questions and summarize the answers suggested by the data, starting with primary outcomes and process outcomes (use).

	1	2	3	4	5	
subitem not at all important	○	○	○	○	◉	essential

Clear selection

Does your paper address subitem 22-i? *

Copy and paste relevant sections from the manuscript (include quotes in quotation marks "like this" to indicate direct quotes from your manuscript), or elaborate on this item by providing additional information not in the ms, or briefly explain why the item is not applicable/relevant for your study

Overall, there were high rates of compliance for a 6-month intensive home monitoring protocol that involved daily EMA ratings of HRQOL, and continuous monitoring of physical activity and sleep by a Fitbit®. Average compliance rates were highest (90%) for activity (step) monitoring (i.e., wearing the Fitbit® during the day), followed by EMA completion (84%), and lowest (75%) for sleep monitoring (i.e., wearing the Fitbit® overnight). In addition, the completion rates for the monthly HRQOL surveys were also high (ranging from 92% to 98%). Compliance and completion rates did not differ by study arm. There was only a moderate relationship between wearing the Fitbit® during the day versus at night, and there was a less robust (i.e., small) relationship between EMA completion rates and Fitbit® compliance rates. These rates are consistent, at the high end, with what has been reported previously in the literature for studies with shorter time durations.1-19 The data on compliance rates for longer study durations in the literature, such as we report from this study, is sparse, but would be expected to be lower than what we found here given typical patterns of decline in compliance rates with longer study duration. Thus, we anticipate the high rates observed in the current study are likely due to the thoughtful use of several study-specific design elements to encourage study engagement. For example, study staff completed regular checks for completion of the EMA questions and monthly surveys, as well as the presence of daily step and sleep data from the Fitbit®. In instances where participants were missing greater than three days' worth of EMA or Fitbit® data, we contacted those participants directly. In addition, automatic reminders for completion of the EMA questions and surveys were sent via the CareQOL app, and study staff contacted participants at least once per month during the home monitoring period to foster engagement. Additionally, we provided monetary compensation for the different elements of the study, including: separate payments of $20 for completing the baseline and 3-month and 6-month post-monitoring period follow-up surveys; $10 compensation for completion of the monthly surveys during the home monitoring period (with the exception of the final monthly survey, for which participants were paid $20); and $1 per day for daily completion of the EMA questions or any daily data from wearing the Fitbit® (either day or night). Participants also could keep the study-provided Fitbit® after they completed the study. Participants were also able to customize what time they received their three EMA questions each day and were offered different wristband options for the Fitbit® to maximize their comfort and style preferences.

In addition to these study design factors that likely influenced compliance, we also were interested in better understanding the participant-specific variables that might have impacted compliance rates. Not surprisingly, we found that positive perceptions about feasibility and acceptability were related to better compliance rates for EMA questions, surveys, steps, and sleep. This finding is consistent with the primary findings of this study, which showed that participants who were more positive about the study itself (regardless of study arm) were more likely to show HRQOL improvements,23 as well as those reported in the general literature that showed compliance rates were higher among participants who found the research study to be more favorable.35, 36 We also found that being Black was associated with lower compliance rates for Fitbit® data (sleep and steps), but not for EMA data. Historically, Black participants are underrepresented in research37, 38 and, consistent with our findings, they have higher rates of missing data than their White counterparts.39-45 Furthermore, being a friend or other family member of the person with TBI (versus a spouse or adult child of the person with TBI) was associated with lower compliance rates for Fitbit® daytime wear data (steps). To our knowledge, while some meta-analytic work has examined [illegible]

caregiver populations (e.g., caregivers of people living with dementia46), this work has not examined caregiver type in consideration of the differential factors that might influence missing data rates. Given this, we postulate that nontraditional caregivers (i.e., friends and other family members) may feel less obligation to provide care than those in more traditional caregiver groups (i.e., spousal and adult child caregivers). Future work to better understand these relationships, how they influence care, and how this may be related to study compliance rates is needed. We also found that being single and/or caring for someone with more functional deficits was associated with lower Fitbit® nighttime wear data (sleep). While we are unaware of work that looks explicitly at these factors and nighttime compliance rates with wearables, we hypothesize that these types of caregivers may already be experiencing fragmented or disturbed sleep and therefore are more likely to find nighttime Fitbit® wear uncomfortable and prohibitive.47

Finally, we explored whether there were meaningful subgroups for different patterns of compliance rates. To this end, we found a "high compliance group-all data" that was compliant with all of the different study elements; a "high compliance group-PROs and steps only" where compliance was high for EMA and survey PROs and Fitbit®-based compliance rates for steps, but not for sleep; and a "moderate PRO compliance, low Fitbit® compliance group" where monthly survey completion was good, but not excellent, and Fitbit® wear-time data was low. These subgroups were predicted by race and relationship to the care recipient, but not to other demographic, clinical, or behavioral characteristics.

22-ii) Highlight unanswered new questions, suggest future research

Highlight unanswered new questions, suggest future research.

	1	2	3	4	5	
subitem not at all important	○	○	○	◉	○	essential

Clear selection

Does your paper address subitem 22-ii?

Copy and paste relevant sections from the manuscript (include quotes in quotation marks "like this" to indicate direct quotes from your manuscript), or elaborate on this item by providing additional information not in the ms, or briefly explain why the item is not applicable/relevant for your study

Teasing apart the differential impact of the different study design elements on study participation will be a focus of future work. Furthermore, consistent with other literature, we found that disadvantaged groups (such as racial/ethnic minorities, single, or non-traditional caregivers) were more likely to have higher rates of missing data than their majority counterparts, further exemplifying the need for more focused work on understanding the reason for these lower rates and employing methods to improve compliance among these groups.

20) Trial limitations, addressing sources of potential bias, imprecision, and, if relevant, multiplicity of analyses

20-i) Typical limitations in ehealth trials

Typical limitations in ehealth trials: Participants in ehealth trials are rarely blinded. Ehealth trials often look at a multiplicity of outcomes, increasing risk for a Type I error. Discuss biases due to non-use of the intervention/usability issues, biases through informed consent procedures, unexpected events.

	1	2	3	4	5	
subitem not at all important	○	○	○	○	◉	essential

Clear selection

Does your paper address subitem 20-i? *

Copy and paste relevant sections from the manuscript (include quotes in quotation marks "like this" to indicate direct quotes from your manuscript), or elaborate on this item by providing additional information not in the ms, or briefly explain why the item is not applicable/relevant for your study

While these results support the feasibility of care partner participation in studies that employ intensive study designs (including EMAs over a 6-month period, wearing of a wrist-worn device that provides continuous monitoring, and completion of end-of-month surveys), it is also important to acknowledge several study limitations. For example, while we have postulated about the reasons the observed completion rates were so high in this study, we did not systematically assess the impact that any of these factors had; future work is needed to explore the impact of factors such as compensation, survey length, etc., on participant completion and compliance rates. In addition, many participants in this sample did not endorse poor HRQOL at baseline, nor high levels of supervision required, nor high levels of assistance with activities required. This could mean that this sample is not experiencing high levels of strain as we had anticipated, and therefore they may have been higher functioning than the general care partner population, such that they had less room for improvement, or they had more capacity/time to complete with the intensive study activities.

21) Generalisability (external validity, applicability) of the trial findings

NPT: External validity of the trial findings according to the intervention, comparators, patients, and care providers or centers involved in the trial

21-i) Generalizability to other populations

Generalizability to other populations: In particular, discuss generalizability to a general Internet population, outside of a RCT setting, and general patient population, including applicability of the study results for other organizations

	1	2	3	4	5	
subitem not at all important	(selected)	○	○	○	○	essential

Clear selection

Does your paper address subitem 21-i?

Copy and paste relevant sections from the manuscript (include quotes in quotation marks "like this" to indicate direct quotes from your manuscript), or elaborate on this item by providing additional information not in the ms, or briefly explain why the item is not applicable/relevant for your study

These limitations have been highlighted in the primary paper: Carlozzi, N.E., Troost, J.P., Sen, S., Choi, S.W., Wu, Z., Miner, J.A., Lombard, W.L., Graves, C., Sander, A.M. (In Press). Improving outcomes for care partners of individuals with traumatic brain injury: Results for a mHealth randomized control trial of the CareQOL app. Archives of Physical Medicine and Rehabilitation.

21-ii) Discuss if there were elements in the RCT that would be different in a routine application setting

Discuss if there were elements in the RCT that would be different in a routine application setting (e.g., prompts/reminders, more human involvement, training sessions or other co-interventions) and what impact the omission of these elements could have on use, adoption, or outcomes if the intervention is applied outside of a RCT setting.

	1	2	3	4	5	
subitem not at all important	(●)	( )	( )	( )	( )	essential

Clear selection

Does your paper address subitem 21-ii?

Copy and paste relevant sections from the manuscript (include quotes in quotation marks "like this" to indicate direct quotes from your manuscript), or elaborate on this item by providing additional information not in the ms, or briefly explain why the item is not applicable/relevant for your study

This is not central to the main findings in this paper

OTHER INFORMATION

23) Registration number and name of trial registry

Does your paper address CONSORT subitem 23? *

Copy and paste relevant sections from the manuscript (include quotes in quotation marks "like this" to indicate direct quotes from your manuscript), or elaborate on this item by providing additional information not in the ms, or briefly explain why the item is not applicable/relevant for your study

Trial Registration: ClinicalTrial.gov NCT04570930; https://clinicaltrials.gov/ct2/show/NCT04570930

24) Where the full trial protocol can be accessed, if available

Does your paper address CONSORT subitem 24? *

Cite a Multimedia Appendix, other reference, or copy and paste relevant sections from the manuscript (include quotes in quotation marks "like this" to indicate direct quotes from your manuscript), or elaborate on this item by providing additional information not in the ms, or briefly explain why the item is not applicable/relevant for your study

Carlozzi NE, Sander AM, Choi SW, et al. Improving outcomes for care partners of persons with traumatic brain injury: Protocol for a randomized control trial of a just-in-time-adaptive self-management intervention. PLoS One. 2022;17(6):e0268726.

25) Sources of funding and other support (such as supply of drugs), role of funders

Does your paper address CONSORT subitem 25? *

Copy and paste relevant sections from the manuscript (include quotes in quotation marks "like this" to indicate direct quotes from your manuscript), or elaborate on this item by providing additional information not in the ms, or briefly explain why the item is not applicable/relevant for your study

Acknowledgements
Work on this manuscript was supported by grant numbers R01NR013658 from the National Institutes of Health (NIH), National Institute of Nursing Research, R01HL146354 and K24HL156896 from the National Heart, Lung and Blood Institute, and UL1TR002240 from the National Center for Advancing Translational Sciences. This work was also supported by the University of Michigan Institute for Healthcare Policy and Innovation. We thank the investigators, coordinators, and research associates/assistants who worked on this study, the study participants, and organizations who supported recruitment efforts.

Site Investigators and Coordinators: Noelle Carlozzi, Sung Won Choi, Zhenke Wu, Srijan Sen, Christopher Graves, Angela Lyden, Nikki Hubbard, Abigail Biddix, Jennifer Miner (University of Michigan, Ann, Arbor, MI); Angelle Sander (Baylor College of Medicine and TIRR Memorial Hermann, Houston, TX), Jay Bogaards (TIRR Memorial Hermann, Houston, TX)

X27) Conflicts of Interest (not a CONSORT item)

X27-i) State the relation of the study team towards the system being evaluated

In addition to the usual declaration of interests (financial or otherwise), also state the relation of the study team towards the system being evaluated, i.e., state if the authors/evaluators are distinct from or identical with the developers/sponsors of the intervention.

	1	2	3	4	5	
subitem not at all important	○	○	◉	○	○	essential

Clear selection

Does your paper address subitem X27-i?

Copy and paste relevant sections from the manuscript (include quotes in quotation marks "like this" to indicate direct quotes from your manuscript), or elaborate on this item by providing additional information not in the ms, or briefly explain why the item is not applicable/relevant for your study

Conflicts of Interest
The authors have no conflicts of interest to report regarding this publication.

About the CONSORT EHEALTH checklist

As a result of using this checklist, did you make changes in your manuscript? *

- ○ yes, major changes
- ○ yes, minor changes
- ◉ no

What were the most important changes you made as a result of using this checklist?

Your answer

How much time did you spend on going through the checklist INCLUDING making changes in your manuscript *

This took me over an hour, and a number of these questions are not framed for secondary analyses of trial data.

As a result of using this checklist, do you think your manuscript has improved? *

- ○ yes
- ◉ no
- ○ Other:

Would you like to become involved in the CONSORT EHEALTH group?

This would involve for example becoming involved in participating in a workshop and writing an "Explanation and Elaboration" document

- ○ yes
- ◉ no
- ○ Other:

Clear selection

Any other comments or questions on CONSORT EHEALTH

Your answer

STOP - Save this form as PDF before you click submit

To generate a record that you filled in this form, we recommend to generate a PDF of this page (on a Mac, simply select "print" and then select "print as PDF") before you submit it.

When you submit your (revised) paper to JMIR, please upload the PDF as supplementary file.

Don't worry if some text in the textboxes is cut off, as we still have the complete information in our database. Thank you!

Final step: Click submit !

Click submit so we have your answers in our database!

Submit

Clear form

Never submit passwords through Google Forms.

This content is neither created nor endorsed by Google. - Terms of Service - Privacy Policy

Does this form look suspicious? Report

Google Forms
